# Endogenous Opioid Antagonism in Physiological Experimental Pain Models: A Systematic Review

**DOI:** 10.1371/journal.pone.0125887

**Published:** 2015-06-01

**Authors:** Mads U. Werner, Manuel P. Pereira, Lars Peter H. Andersen, Jørgen B. Dahl

**Affiliations:** 1 Multidisciplinary Pain Center, Neuroscience Center, Rigshospitalet, Copenhagen, Denmark; 2 Department of Anaesthesia, Centre of Head and Orthopaedics, Rigshospitalet, Copenhagen, Denmark; 3 Department of Surgery D, Herlev Hospital, Copenhagen, Denmark; University of British Columbia, CANADA

## Abstract

Opioid antagonists are pharmacological tools applied as an indirect measure to detect activation of the endogenous opioid system (EOS) in experimental pain models. The objective of this systematic review was to examine the effect of mu-opioid-receptor (MOR) antagonists in placebo-controlled, double-blind studies using ʻinhibitoryʼ or ʻsensitizingʼ, physiological test paradigms in healthy human subjects. The databases PubMed and Embase were searched according to predefined criteria. Out of a total of 2,142 records, 63 studies (1,477 subjects [male/female ratio = 1.5]) were considered relevant. Twenty-five studies utilized ʻinhibitoryʼ test paradigms (ITP) and 38 studies utilized ʻsensitizingʼ test paradigms (STP). The ITP-studies were characterized as conditioning modulation models (22 studies) and repetitive transcranial magnetic stimulation models (rTMS; 3 studies), and, the STP-studies as secondary hyperalgesia models (6 studies), ʻpainʼ models (25 studies), summation models (2 studies), nociceptive reflex models (3 studies) and miscellaneous models (2 studies). A consistent reversal of analgesia by a MOR-antagonist was demonstrated in 10 of the 25 ITP-studies, including stress-induced analgesia and rTMS. In the remaining 14 conditioning modulation studies either absence of effects or ambiguous effects by MOR-antagonists, were observed. In the STP-studies, no effect of the opioid-blockade could be demonstrated in 5 out of 6 secondary hyperalgesia studies. The direction of MOR-antagonist dependent effects upon pain ratings, threshold assessments and somatosensory evoked potentials (SSEP), did not appear consistent in 28 out of 32 ʻpainʼ model studies. In conclusion, only in 2 experimental human pain models, i.e., stress-induced analgesia and rTMS, administration of MOR-antagonist demonstrated a consistent effect, presumably mediated by an EOS-dependent mechanisms of analgesia and hyperalgesia.

## Introduction

Human experimental pain models are essential in physiological and pharmacological research, testing hypothetical pain mechanisms, forward-translating observations from animal research or establishing evidence of analgesic drug efficacy. A number of receptor-specific agonists and antagonists are utilized as adjuncts investigating physiologic mechanisms behind pain inhibition and pain sensitization. Research has focused on various receptors, e.g., α_2_-receptors, 5-HT_1A_-receptors, NMDA-receptors and TRPV1-receptors, but above all, major interest has been dedicated to the endogenous mu-opioid-receptor (MOR). Selective MOR-antagonists have been used in a large number of human experimental [1–63] and clinical studies [64]. Early animal data demonstrated that MOR-antagonists increase nociceptive responding across various stimulation paradigms and species [61]. Subsequent studies in monkeys and humans showed that microinjections of morphine [65] or electrical stimulation [66] of the periaqueductal grey area (PAG) produced marked analgesia, which could effectively be antagonized by systemic administration of naloxone [67].

In human experimental pain models the research involving MOR-antagonists has primarily focused on pain thresholds and tolerance to pain stimuli, conceptualizing the idea that activity of the EOS hypothetically could be responsible for an attenuation of the responses to pain [43]. Consequently the administration of MOR-antagonist could indirectly substantiate or question the involvement of the EOS in acute experimental pain perception. Since results from the literature on the effect of MOR-antagonists on experimental pain seem ambiguous [57,61], the authors decided to undertake a systematic review separating the search data into studies utilizing ʻinhibitoryʼ test paradigms and ʻsensitizingʼ test paradigms. The main objective was to examine if certain physiological stimulation paradigms, techniques or methods could be modulated by naloxone or naltrexone, which is considered presumptive evidence of activation of the EOS. The primary outcomes were direct measures of experimental pain perception (pain ratings, pain thresholds, pain tolerance, hyperalgesia) or indirect measures of nociception (neuroimaging responses [BOLD (blood-oxygen-level dependent) contrast imaging, fMRI, PET], nociceptive reflexes [NRF], somatosensory evoked potentials [SSEP]). The secondary outcomes were autonomic measures of pain and nociception (autonomic, hemodynamic and neuroendocrine responses).

## Materials and Methods

### 2.1 Registration and Search Strategy

The review was registered in the PROSPERO international database (CRD42014013102; http://www.crd.york.ac.uk/PROSPERO/DisplayPDF.php?ID=CRD42014013102). Only placebo-controlled, double-blind, experimental studies, including healthy human subjects, examining the effect of MOR-antagonists on pain inhibition and pain sensitization, were considered. It was required that the studies employed physiological stimuli, i.e., chemical, electrical, mechanical, pharmacological, thermal or a combination of stimuli. Psychological conditioning stimuli, often applied in placebo or behavioral studies, were not included in this review. Studies primarily concerning acupuncture, cardiovascular reactivity, clinical outcomes, endocrine functions, psychological or psychiatric outcomes and substance abuse, as well as, non-English studies, abstracts from scientific meetings and material from textbooks were not included. Studies with opioid-administration prior to administration of the MOR-antagonist were not included.

**Fig 1:**
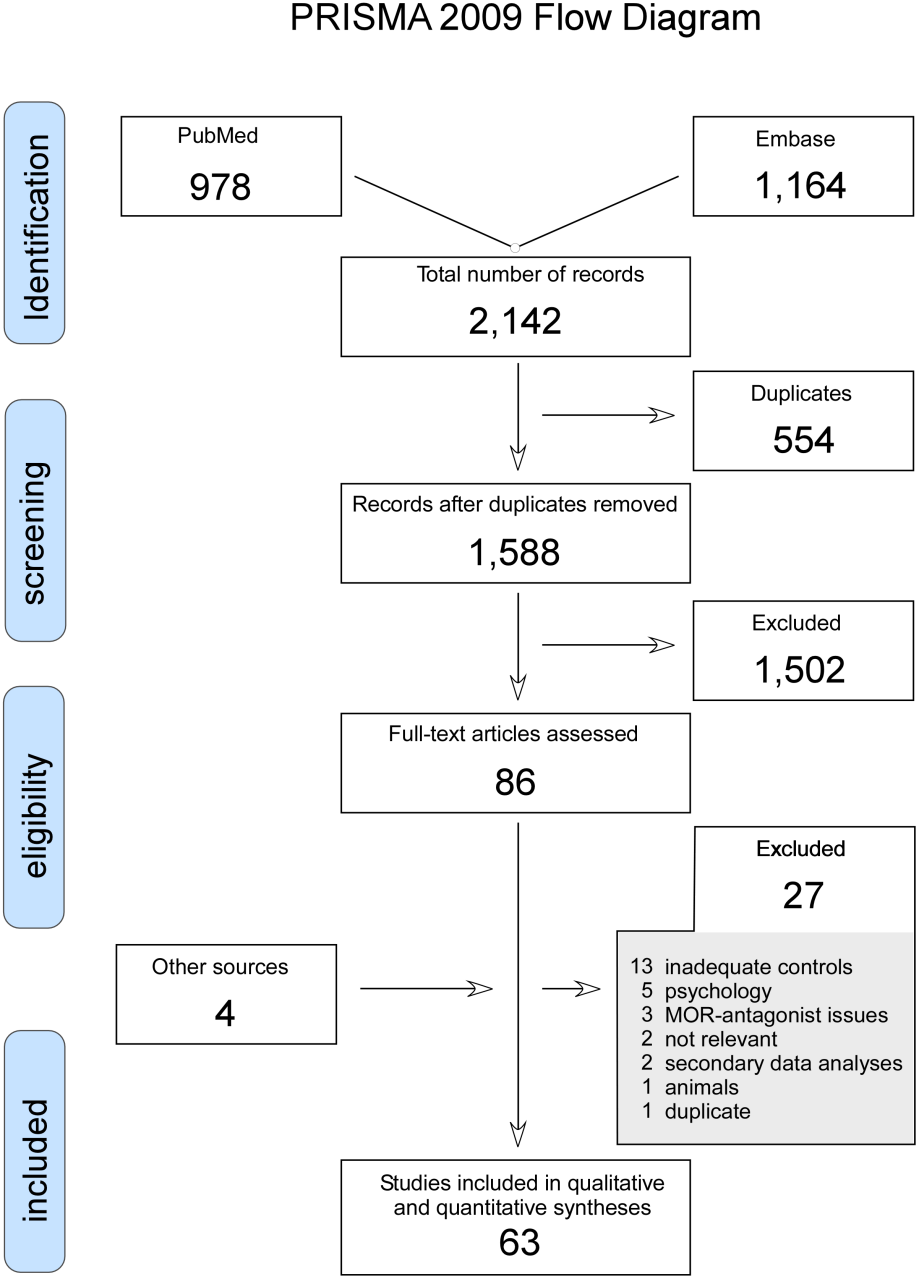
The search algorithm according to the PRISMA-requirements [126].

A literature search (LPHA, MPP, MUW) was performed in the databases PubMed and EMBASE (search completed August 8, 2014) using the following search terms: (pain OR pain measurement OR pain threshold OR pain perception OR pain sensitization OR pain inhibition OR pain summation OR pain conditioning OR pain habituation OR pain modulation OR secondary hyperalgesia OR hyperalgesia OR diffuse noxious inhibitory controls OR diffuse noxious inhibitory control OR DNIC) AND (levallorphan OR naloxone OR naltrexone OR methyl-naltrexone OR alvimopan OR diprenorphine OR meptazinol OR Receptors, Opioid, mu/antagonists and inhibitors OR mu-opioid receptor antagonist OR mu opiate receptor antagonist) AND (healthy OR subjects OR control group OR normal OR normals OR double-blind placebo controlled OR double-blind method). Reference-lists from retrieved studies were searched for additional relevant material (MUW). No contact with study authors to identify additional studies was made. In case of uncertainty concerning relevance of an article, the subject was discussed between the authors and a final decision was taken by the senior author (MUW). From the 2,142 records 86 full-text articles were assessed for eligibility. Sixty-three relevant studies were included in the review (Fig 1: PRISMA 2009 Flow Diagram). Assessing risk of bias was made by the Oxford quality scoring system [68] (MPP, MUW). Descriptive data and outcome data were extracted from these studies and accumulated in tables (MUW) and verified independently (MPP, LPHA). The PRISMA 2009 Checklist is in a supporting file (S1 PRISMA Checklist).

### 2.2 Definitions

Preliminary examination of the retrieved studies indicated that a classification of the studies into ʻinhibitoryʼ and ʻsensitizingʼ test paradigms would facilitate the presentation and interpretation of data.

#### 2.2.1 ʻInhibitoryʼ Test Paradigms (ITP)

**Fig 2:**
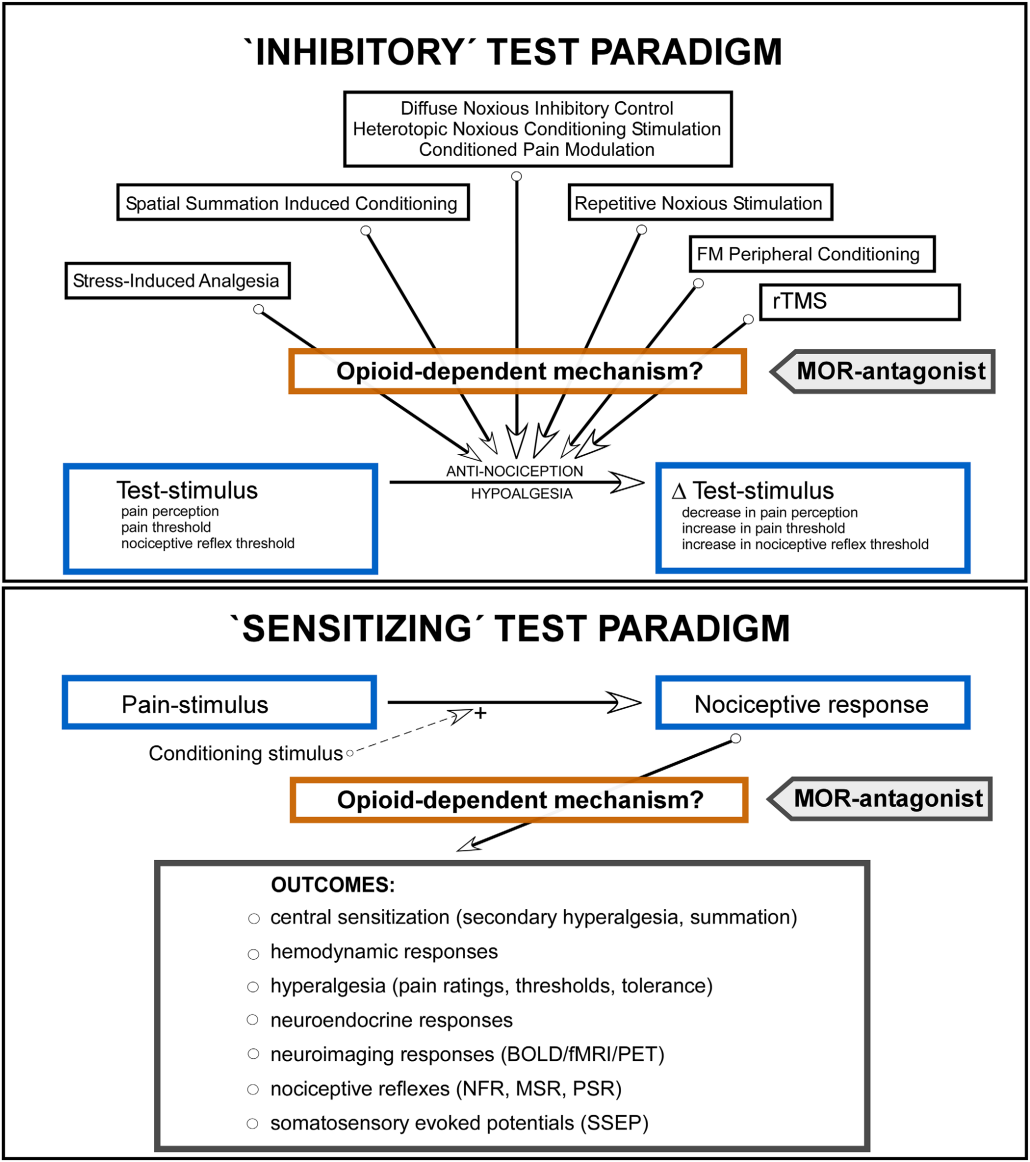
Schematic illustration of the ʻinhibitoryʼ test paradigms (ITP, upper panel) and the ʻsensitizingʼ test paradigms (STP, lower panel). The ITP-studies employed an inhibitory conditioning stimulus with evaluation of the associated change in the applied test-stimulus (△test-stimulus). The objective of the ITP-studies was to examine the effect of mu-opioid-receptor (MOR) antagonist on the magnitude of the △test-stimulus, indicating an activation of the endogenous opioid system (EOS) responsible for the conditioning response leading to antinociception/hypoalgesia (the central rectangle [Opioid-dependent mechanism?] indicates a hypothetical augmentation of the conditioning response by the EOS). The STP-studies (lower panel) employed a pain stimulus leading to quantifiable ʻsensitizingʼ CNS-responses, e.g., changes in behavioral measures (hyperalgesia, pain ratings, thresholds, tolerance), nociceptive reflexes, neuroimaging or neuroendocrine variables. In a number of studies a sensitizing conditioning stimulus was applied, e.g., a burn injury [31] and application of capsaicin [35,36], enhancing the nociceptive responses. The objective of the STP-studies was to examine the effect of MOR-antagonist on the magnitude of elicited responses, indirectly either supporting or contradicting an effect mediated by the EOS (the central rectangle [Opioid-dependent mechanism?] indicates a hypothetical attenuation of the response by the EOS). FM Peripheral Conditioning = non-noxious Frequency Modulated Peripheral Conditioning; rTMS = repetitive Transcranial Magnetic Stimulation.

ITP-studies were characterized by implementation of a noxious or non-noxious inhibitory conditioning stimulus (Fig 2, upper panel; stress-induced analgesia [SIA], spatial summation induced conditioning, diffuse noxious inhibitory control [DNIC], heterotopic noxious conditioning stimulation, conditioned pain modulation [CPM], repetitive noxious stimulation, non-noxious frequency modulated peripheral conditioning and repetitive transcranial magnetic stimulation [rTMS]) [69]. The test-stimulus (Fig 2) was applied heterotopically, at a site different from the site of the conditioning stimulus, or homotopically, at the same site as the conditioning stimulus, where the test stimulus became an integrated part of the conditioning stimulus [19]. The response to the test-stimulus was evaluated by psychophysical measures, e.g., pain ratings, pain threshold and pain tolerance assessments, or physiological measures, e.g., the spinal nociceptive flexion reflex (RIII; Fig 2) [70]. The conditioning inhibitory effect was evaluated by the associated decrease in the response to the test-stimulus: △test-stimulus (Fig 2). MOR-antagonist was administered in order to indirectly uncover an EOS-dependent mechanism in the conditioning response: if the △test-stimulus was attenuated by the MOR-antagonist, a role of the EOS was presumed. In all the studies the outcomes were evaluated against baseline conditions and placebo-controls.

#### 2.2.2 ʻSensitizingʼ Test Paradigms (STP)

STP-studies were characterized by implementation of a pain stimulus leading to quantifiable, ʻsensitizingʼ, nociceptive responses, i.e., changes in behavioral measures (hyperalgesia, pain ratings, thresholds, pain tolerance), thresholds of nociceptive reflexes, SSEP, or, miscellaneous neuroimaging or neuroendocrine variables (Fig 2, lower panel). In a number of the STP-studies an additional conditioning stimulus was applied, e.g., a burn injury [31] or capsaicin [35,36], enhancing the nociceptive response. MOR-antagonists were administered in order to indirectly uncover an EOS-dependent mechanism in the ʻsensitizingʼ nociceptive response: if the response was enhanced by the MOR-antagonist, an inhibitory role of the EOS was presumed. In all the studies the outcomes were evaluated against baseline conditions and placebo controls.

#### 2.2.3 Habituation and Sensitization

The phenomenon by which repeated identical stimuli elicit progressively decrements in responses has been operationally defined as habituation [71]. The phenomenon by which repeated identical stimuli elicit progressively increments in responses is here defined as sensitization.

### 2.3 MOR-antagonists

The MOR-antagonists used in human research are alvimopan, diprenorphine, methylnaltrexone, naloxone and naltrexone. In addition, MOR-antagonists, or MOR-antagonists with partial κ-agonist effects, levallorphan, meptazinol and nalorphine, have been used in opioid blocking research. In the retrieved ITP- and STP-studies only naloxone and naltrexone were used.

Naloxone and naltrexone are non-specific opioid-antagonists with high affinity for the MOR [72]. Both drugs cross the blood-brain barrier and demonstrate central opioid-blocking effects, in contrast to the peripherally acting MOR-antagonists, e.g., alvimopan and methyl-naltrexone. Due to low systemic bioavailability of naloxone after oral administration, i.e., 2–3% [73], naloxone is given parenterally, when systemic opioid-blocking effects are required. In adults the distribution half-life (T_½α_) is 40 to 70 seconds [74], and the elimination (T_½β_) half-life is 54 to 64 min [74,75]. Naloxone, with a rapid onset and short duration of action, is suited for acute management of opioid-induced serious adverse effects [24] and is administered in IV doses of 0.04 mg to 0.4 mg [76]. Interestingly, naloxone expresses a dose-dependent, biphasic response with low doses producing analgesia and high doses producing hyperalgesia, both in animal inflammatory models [77] and in clinical models [78–80].

Naltrexone has a systemic bioavailability after oral administration of 5% to 60% [81] and since its main use clinically is treatment of substance dependence, the oral route is preferred. The elimination half-life of naltrexone and its active metabolite 6-beta-naltrexol, after oral administration is 4 to 10 hours [82]. Naltrexone is clinically given in daily doses of 50 to 100 mg.

## Results

### 3.1 Literature Search

The search algorithm with the number of retrieved studies is presented in Fig 1. A total of 2,142 records were retrieved, and after subtracting 554 repeat entries, 1,588 records were considered for analysis. From these 1,502 records were not considered relevant for the review and therefore excluded. Eighty-six full text articles were assessed and of these 27 were excluded. Four additional studies were retrieved from reference lists and from consultation with experts in the field giving a total of 63 studies considered relevant for this review [1–63].

### 3.2 Research Areas

For the sake of clarity, data for ITP and STP are presented separately, each in a subsection.

#### 3.2.1 ʻInhibitoryʼ Test Paradigms (25 studies)

The research areas were conditioning modulation models (22 studies) [1–22] and rTMS-models (3 studies) [23–25].

#### 3.2.2 ʻSensitizingʼ Test Paradigms (38 studies)

The research areas were secondary hyperalgesia models (6 studies) [26–31], summation models (2 studies) [32,33], ʻpainʼ models (25 studies) [34–58], nociceptive reflex models (3 studies) [59–61] and miscellaneous (2 studies) [62,63].

### 3.3 Study Design

#### 3.3.1 ʻInhibitoryʼ Test Paradigms

Study designs are presented in Table 1. All studies were double-blind and placebo-controlled, and, 17 of the 25 studies [1,2,6,8–10,12,14–17,20–25] were randomized. Four studies reported a counter-balanced design [6,9,20,21], while 19 studies reported use of a cross-over design [1,2,6,7,10–18,20–25]. Three studies, investigating rTMS-induced analgesia, used a sham-control [23–25]. One study used a control 25°C water-immersion test [21].

**Table 1:** ʻInhibitoryʼ Test Paradigms: Study Design.

[Ref.] First Author	Year	Study Design	Primary Objective^△^	Secondary Objective^△^	Oxford Quality Score
ʻINHIBITORYʼ TEST PARADIGMS					
Conditioned Modulation Models					
[1] Willer JC	1980	DB, R, PC, 3-WX	Effect of Nx on stress-induced analgesia	NRR	2
[2] Willer JC	1981	DB, R, PC, 3-WX	Effect of Nx in stress-induced analgesia: pain reflexes	Effect of naloxone on development of hyperalgesia	2
[3] Pertovaara A	1981	DB, PC	Effect of Nx on dental pain threshold during non-painful high-frequency TENS	NRR	1
[4] Pertovaara A	1982	DB, PC	Effect of Nx on ischemia-induced pain reduction	Effect of naloxone on ischemia-induced changes in thermal sensitivity	2
[5] Willer JC	1982	DB, PC,	Effect on Nx on the depression on the nociceptive blink reflex induced by high frequency conditioning stimulation	NRR	1
[6] Pertovaara A	1982	DB, R, CB, PC, 2-WX	Effect of Nx on dental pain threshold during non-painful low-frequency TENS	NRR	1
[7] Buchsbaum MS	1983	DB, PC, 3-WX	Effect of Nx on pain sensitivity (assessed by EPs) before and after repeated electrical stimulation	NRR	2
[8] Jungkunz G	1983	DB, R, PC, 4-PG	Effect of Nx on cold pressor induced changes in electrical pain thresholds	Effect of naloxone on mentally stress induced changes in electrical pain thresholds	3
[9] Janal M	1984	DB, R, PC, CB	Effect of Nx on thermal and ischemic responses after exercise	NRR	2
[10] Willer JC	1986	DB, R, PC, 4-WX	Effect of Nx on stress-induced changes in nociceptive flexion reflex threshold	NRR	2
[11] Ernst M	1986	DB, PC, 2-WX	Effect of Nx on habituation to repeated noxious stimuli	NRR	1
[12] Willer JC	1986	DB, R, PC, 4-WX	Effect of Nx on habituation to repeated stress stimuli	Effect of naloxone on autonomic parameters during repeated stress stimuli	2
[13] Olausson B	1986	DB, PC, 2-WX, 4-SX	Effect of Nx on dental pain thresholds following low-frequency TNS	Effect of Nx on dental pain thresholds following muscular exercise	2
[14] Willer JC	1990	DB, R, PC, 2-WX	Effect of Nx on DNIC assessed by the nociceptive flexion reflex	NRR	2
[15] Poulsen L	1996	DB, R, PC, 2-WC, 4-SX	Effect of Nx on DNIC assessed by the nociceptive flexion reflex in extensive and poor metabolizers of sparteine (CYP2D6)	Effect of Nx on pain ratings during cold pressor test in extensive and poor metabolizers of sparteine (CYP2D6)	2
[16] Edwards RR	2004	DB, R, PC, 2-WX	Effect of Nx on DNIC	Effect of Nx on the relationship of cardiovascular reactivity and DNIC	2
[17] Julien N	2006	DB, R, PC, 4-WX	Effect of Nx on spatial summation induced activation of endogenous pain inhibitory system	NRR	2
[18] Robertson LJ	2008	DB, PC, 2-WX	Local effect of Nx on thermal hyperalgesia after a burn injury modified by repeated cold water immersions	NRR	1
[19] Rennefeld C	2010	DB, PC, 8-S	Effect of Nx on habituation to repeated pain stimuli	NRR	1
[20] Leonard G	2010	DB, R, PC, CB, 3-WX, 2-D	Effect of Nx on high-frequency TENS	NRR	3
[21] Sprenger C	2011	DB, R, PC, 2-WX, CB	Effect of Nx on HNCS^¤^ induced by CWIT and evaluated by behavioral responses	Effect on Nx on HNCS^¤^ induced by CWIT and evaluated by BOLD responses	3
[22] King CD	2013	DB, R, PC, 2-WX	Effect of NTx on CPM	NRR	3
Repetitive Transcranial Magnetic Stimulation Models					
[23] de Andrade DC	2011	DB, R, PC, SC, 2-WX, 3-PG	Effect of Nx on DLPFC/PMC- and M1-targeted repetitive transcranial magnetic stimulation induced analgesia	NRR	3
[24] Taylor JJ	2012	DB, R, PC, SC, 2x2-WX	Effect of Nx on LDPFC-targeted repetitive transcranial magnetic stimulation induced analgesia	NRR	4
[25] Taylor JJ	2013	DB, R, PC, SC, 2x2-WX	Effect of Nx on LDPFC-targeted repetitive transcranial magnetic stimulation induced analgesia	NRR	5

^△^ Objectives related to the specific perspectives of the review.

^¤^ HNCS in man, DNIC in animals (the authors’ terminology [21]).

**BI** = first-degree burn injury; **BOLD** = blood-oxygen-level dependent contrast imaging; **BTS** = brief thermal sensitization; **CB** = counterbalanced; **CPTT** = cold pressor test; **DB** = double-blind; **CYP2D6** = cytochrome P450 2D6 enzyme; **CPM** = conditioned pain modulation; **DLPFC/PMC** = right dorsolateral–prefrontal cortex premotor cortex (see **LDPFC**); **DNIC** = diffuse noxious inhibitory controls; **EP** = [somatosensory] evoked potentials; **EPT** = electrical pain threshold; **EPIS** = endogenous pain inhibitory system; **fMRI** = functional magnetic resonance imaging; **HNCS** = heterotopic noxious conditioning stimulations; **IDES** = intradermal electrical stimulation (rectangular, 0.5 ms duration, 2 Hz, high density); **LDPFC** = left dorsolateral prefrontal cortex; **M1** = primary motor cortex; **NFR** = nociceptive flexion reflex; **NRR** = not relevant for the review; **NTx** = naltrexone; **Nx** = naloxone; **OIH** = opioid-induced hyperalgesia; **R** = randomized; **PC** = placebo-controlled; **SB** = single-blind; **SBP** = systolic blood pressure; **SC** = sham-controlled; **SHA** = secondary hyperalgesia area; **SOWS** = subjective opioid withdrawal scale; **SSEP** = EP; **TCI** = target-controlled infusion; **TDES** = transdermal electrical stimulation (low density); **TENS** = transcutaneous electrical nerve stimulation; **X** = cross-over (side to side); **2-D/3-D** = two-/two-dose; **2-WX /3-WX/4-WC** = two-/three-/four-way cross-over; **3-SX/4-SX/5-SX/6-SX** = three-/four-/five-/six-session cross-over study; **8-S** = eight session study; **2-PG/3-PG/4-PG/6-PG** = two/three/four/six parallel-groups; **2x2-WX** = 2 parallel groups each with a 2 way-cross over design.

#### 3.3.2 ʻSensitizingʼ Test Paradigms

Study designs are presented in Table 2. All of the studies were placebo-controlled while 37 of the 38 studies were double-blind [26–37,39–63]. However, one study [37] mentions only blinding of the subjects, but the study is registered as a controlled clinical trial. Thirty of these studies were randomized [26–32,34–36,39–42,44–52,54–56,58,60,61,63], while 12 studies [35,37,38,41–43,48,50–53,57] used a counter-balanced design. Eight studies did not report a randomized design [33,37,38,43,53,57,59,62].

**Table 2:** ʻSensitizingʼ Test Paradigms: Study Design.

[Ref.] First Author	Year	Study Design	Primary Objective^△^	Secondary Objective^△^	Oxford Quality Score
ʻSENSITIZINGʼ TEST PARADIGMS					
Secondary Hyperalgesia Models					
[26] Mikkelsen S	1999	DB, R, PC, 3-WX	Effect of pre-emptive Nx on ketamine-induced SHA	NRR	5
[27] Brennum J	2001	DB, R, PC, 3-WX, 2-D	Effect of Nx on SHA induced by BI	NRR	3
[28] Koppert W	2003	DB, R, PC, 4-WX	Effect of Nx on SHA/pain induced by IDES/TDES	NRR	2
[29] Koppert W	2005	DB, R, PC, ^†^ 4-SX, TCI	Effect of Nx on SHA/pain induced by IDES	NRR	2
[30] Chu FL	2011	DB, R, PC, 2-WX^#^	Effect of Nx on OIH induced by remifentanil	Effect of Nx on SHA/pain induced by IDES	3
[31] Pereira MP	2013	DB, R, PC, 2-WX	Effect of Nx on reinstatement of SHA induced by BI	Effect of Nx on SHA induced by BTS	5
Summation Models					
[32] Benedetti F	1999	DB, R, PC, 6-PG	Effect of Nx on spatially directed expectation of pain	NRR	2
[33] Price DD	2002	DB, PC, 4-PG	Effect of Nx on heat- and cold-induced temporal summation of second pain	Effect of Nx on first and second pain	1
ʻPainʼ Models					
*Capsaicin*:					
[34] Graven-Nielsen	2002	DB, R, PC, 2-WX	Effect of Nx on capsaicin-induced muscle pain	NRR	2
*Capsaicin & heat*:					
[35] Drummond PD	2000	CB, R, PC, 2-WX	Effect of iontophoretically applied Nx on capsaicin induced heat-sensitization	NRR	1
[36] Anderson WS	2002	DB, R, PC, 2-WX	Effect of Nx on capsaicin-induced pain kindled by heating	NRR	3
*Comb*. *modalities*, *others*:					
[37] Grevert P	1978	DB, CB, PC, 3-WX, 2-D, 2-PG	Effect of Nx on pain induced by ischemia and cold-water immersion	NRR	1
[38] McCubbin JA	1994	SB, CB, PC, 2-WX	Effect of Nx on pain rating for hand-grip challenge and cold pressor challenge	Effect of Nx on relationship between SBP and pain ratings.	1
[39] Stacher G	1988	DB, R, PC, 3-WX, 2-D	Effect of Nx on threshold and tolerance to electrically induced pain and threshold to heat-induced pain	NRR	3
[40] Younger JW	2009	DB, R, PC, 2-WX	Effect of NTx on changes in sensitivity to heat, cold, and mechanical pain	Effect of NTx on mood and opioid-withdrawal symptoms (SOWS)	5
[41] Bruehl S	2012	DB, R, PC, CB, 2-WX	Effect of Nx used as tool revealing endogenous opioid activity during ischemic and pressure pain tests	NRR	2
[42] Bruehl S	2013	DB, R, CB, PC, 3-SX	Effect of Nx used as tool revealing endogenous opioid activity during ischemic and heat pain tests	NRR	3
*Electrical*					
[43] El-Sobky A	1976	DB, CB, PC, 3-WX, 2-D	Effect of Nx on electrically induced pain threshold and tolerance	NRR	1
[44] Buchsbaum MS	1977	DB, R, PC, 2-WX	Effect of Nx on pain sensitivity after low to high intensity electrical stimulation	Effect of Nx on SSEP after low to high intensity electrical stimulation	3
[45] Bromm B	1983	DB, R, PC, 5-SX	Effect of Nx on pain sensitivity to phasic electrical stimuli	Effect of Nx on pain SSEP after single repeated electrical stimuli	3
*Ischemia*:					
[46] Grevert P	1977	DB, R, PC, 3-WX, 2-D	Effect of Nx on pain induced by the tourniquet test	NRR	3
[47] Grevert P	1983	DB, R, PC, 3-WX, 2-D	Effect of an 8 hr Nx-infusion on pain induced by the tourniquet test	Effect of 8 hr Nx-infusion on cortisol, β-endorhin and blood pressure	2
[48] Posner J	1985	DB, R, CB, PC, 6-SX	Effect of Nx on pain induced during the tourniquet test	NRR	3
*Mechanical*:					
[49] Schobel HP	1998	DB, R, PC, 2-WX	Effect of Nx on pain ratings to pinching stimuli	Effects of Nx on hemodynamic and sympathetic responses to pain	2
[50] Cook DB	2000	DB, R, CB, PC, 3-WX	Effect of NTx on pain induced by dynamic hand grip fatiguing exercise	Effect of NTx on sympathetic nerve activity during exercise	3
*Thermal*:					
[51] Lautenbacher S	1990	DB, R, CB, PC, 2-WX	Effect of Nx on pain induced by tonic and phasic heat stimuli	NRR	2
[52] Lautenbacher S	1994	DB, R, CB, PC, 2-WX	Effect of Nx on heat and cold pain thresholds, and vibratory thresholds	NRR	2
[53] Al’Absi M	2004	DB, CB, PC, 2-WX	Effect of NTx on pain induced by heat and CPTT	NRR	2
[54] Borras MC	2004	DB, R, PC, 2-WX	Effect of Nx on pain and CNS-responses (fMRI) to suprathreshold heat stimuli		2
[55] Kern D	2008	DB, R, PC, 2x2-WX,	Effect of Nx on paradoxical pain induced by the “thermal grill”	Effect of Nx on thermal thresholds	4
[56] Kotlyar M	2008	DB, R, PC, 2-WX	Effect of NTx on pain induced by CPTT	Effect of NTx on sympathetic responses induced by CPTT	3
[57] Schoell ED	2010	DB, CB, PC, 2-WX	Effect of Nx on pain ratings and CNS-responses (BOLD) to suprathreshold heat stimuli		2
[58] Pickering G	2013	DB, R, PC, 4-WX	Effect of Nx on pain induced by repeated heat stimuli	Effect of Nx on SSEP induced by heat	5
Nociceptive Reflex Models					
[59] Boreau F	1978	DB, PC	Effect of Nx on spinal reflexes	NRR	1
[60] France CR	2005	DB, R, PC, 2-WX	Effect of NTx on pain ratings, NFR thresholds and EPT assessments.	NRR	3
[61] France CR	2007	DB, R, PC, 2-WC	Effect of NTx on pain thresholds, pain tolerance and NFR recordings.	NRR	3
Miscellaneous Models					
[62] Eissenberg T	2000	DB, PC, 4-WX	Effect of NTx on reversal of oxycodone induced antihyperalgesia in UV-exposed skin	NRR	2
[63] Robertson LJ	2007	DB, R, PC, X	Local effect of Nx on opioid induced antihyperalgesia following a burn	NRR	2

^△^ Objectives related to the specific perspectives of the review.

^†^ ratio of placebo-treated vs. naloxone-treated was 0.5.

^#^ study design is for remifentanil-placebo infusions.

For explanation of abbreviations, please refer to legend Table 1.

### 3.4 Quality Scoring

#### 3.4.1 ʻInhibitoryʼ Test Paradigms

Evaluation was by the Oxford quality scoring system [68] (Table 1). The median (25–75% IQR) score was 2 (2 to 3). Seven out of 25 studies qualified for a score > 2 [8,20–25] and 6 studies for a score < 2 [3,5,6,11,18,19]. In 5 studies either the randomization [22,25] or the blinding procedure [7,13,24,25] was described, but in the remaining 20 studies no information on these procedures were presented. In 5 studies withdrawals and the reasons for withdrawing subjects were reported [8,13,21,24,25].

#### 3.4.2 ʻSensitizingʼ Test Paradigms

Evaluation was by the Oxford quality scoring system (Table 2) [68]. The median (25–75% IQR) score was 2 (2 to 3). Eighteen of 38 studies qualified for a score > 2 [26,27,30,31,36,39,40,42,44–46,48,50,55,56,58,60,61] and 6 studies for a score < 2 [33,35,37,38,43,59]. In 6 [26,31,39,40,45,58] and 10 [26,31,40,42,46,48,50,55,56,58] studies, respectively, the randomization or the blinding procedure was described. In 26 studies no information on these procedures were presented [27–30,32–38,41,43,44,47,49,51–54,57,59–63]. However, 16 studies reported withdrawals and the reasons for withdrawing subjects [26,27,30,31,36–38,40,44,53,54,57,58,60–62].

### 3.5 Statistics

#### 3.5.1 ʻInhibitoryʼ Test Paradigms

None of the 25 studies reported *a priori* sample size estimations. In 5 studies the confounding issue of limited sample size was discussed [15,16,18,20,22]. Effect size calculations with estimates of Cohen’s *d* and partial η^2^ (eta squared) [83] were reported in 2 studies [16,22]. In 3 studies corrections for multiple comparisons were made with the Bonferroni adjustment [24,25] and the Tukey-Kramer method [23], respectively. Association was estimated by the Pearson’s correlation coefficient (*r*) in 10 studies [1,2,4,6,11,14,16,18,21,22]. Analyses of variance (one-way/two-way/three-factor/repeated measures/mixed model ANOVAs) [7,9,12,14,16–19,22–25] or covariance [11] were performed in 13 out of the 25 studies.

#### 3.5.2 ʻSensitizingʼ Test Paradigms

*A priori* sample size estimations were reported in 4 [27,31,40,50] of the 38 studies. *Post-hoc* sample size estimates [40] including analyses with Fisher’s *post-hoc* least significant difference (LSD) [49,55] were made in 3 studies. In 5 studies the issue of limited sample size was discussed [40,41,50,56,63]. Effect size calculations with estimates of Cohen’s *d* and partial η^2^ were reported in 2 studies [33,50] and with correlation coefficients in 1 study [41]. In 10 studies corrections for multiple comparisons were made with Bonferroni, Newman-Keul’s multiple range test, Scheffés *post-hoc* test, Tukey’s test or by applying a 1% significance level [26,28,29,31,32,34,35,48,62,63]. Analyses of variance (one-way/two-way/three-factor/repeated measures ANOVAs; multivariate ANOVA [MANOVA; WILKS test]; linear mixed models; Friedman test) [27–29,32–36,38–40,42,44–53,55–58,60,62,63] were performed in 29 out of 38 studies. Association was estimated by Pearson’s correlation coefficient (*r*) in 7 studies [28,42,44,45,50–52] and by logistic regression analyses in 1 study [41]. Multiple regression analyses with general linear models (GLM) were made in 2 studies [38,54]. Estimation of significance of indirect effects was made by the Sobel test and by bootstrap estimates [84] in 1 study [42]. Calculations compensating for extreme outliers by Winsorized blockade effect measures were made in 1 study [41].

### 3.6 Demographics

#### 3.6.1 ʻInhibitoryʼ Test Paradigms

Demographics are presented in Table 3. The total number of subjects in the ITP-studies was 429, with a median (IQR) number of subjects in each study of 14.0 (8.0 to 24.0). Two studies did not report the gender of the subjects [8,25], but calculated from the remaining 23 studies, the gender ratio (males/females) was 1.9 (249/134). Interestingly, none of the studies rendered information concerning body weight, a detail of some importance, since 11 of the studies used weight-based infusion regimens [5,10,12,16,17,19–21,23–25].

**Table 3:** ʻInhibitoryʼ Test Paradigms: Demographics and Drugs.

[Ref.] First Author	N	Male/Female	Age (yr)	Drug	Dose	Administration	Additional drugs^§^
ʻINHIBITORYʼ TEST PARADIGMS							
Conditioned Modulation Models							
[1] Willer JC	6	4/2	Range: 23–24	Nx	B: 4 mg	i.v.	-
[2] Willer JC	6	4/2	Range: 22–35	Nx	B: 5 mg	i.v.	-
[3] Pertovaara A	6	6/0	Range: 23–37	Nx	B: 0.8 mg	i.v.	-
[4] Pertovaara A	10^#^	10/0	Range: 20–38^A^	Nx	B: 2 mg	i.v.	-
[5] Willer JC	15	10/5	Range: 21–33	Nx	B: 0.02 mg/kg	i.v.	-
[6] Pertovaara A	7	6/1	Range: 21–27	Nx	B: 0.8 mg	i.v.	-
[7] Buchsbaum MS	19	10/9	NR	Nx	B: 8 mg	i.v.	
[8] Jungkunz G	32	NR	NR	Nx	B: 0.8 mg	i.v.	-
[9] Janal M	12	12/0	Mean: 39 ± 12^SD^	Nx	B: 0.8 mg	i.v.	-
[10] Willer JC	8	4/4	Range: 26–38	Nx	B: 0.06–0.07 mg/kg	i.v.	Diazepam
[11] Ernst M	6	2/4	NR	Nx	B: 1.2 mg	i.m.	-
[12] Willer JC	8	4/4	Range: 25–36	Nx	B: 0.08 mg/kg	i.v.	Diazepam
[13] Olausson B	11	8/3	Range: 21–40	Nx	B: 0.8 mg	i.v.	-
[14] Willer JC	9	4/5	Range: 23–36	Nx	B: 0.4 mg	i.v.	-
[15] Poulsen L	41	26/15	NR	Nx	B: 0.8 mg	i.v.	-
[16] Edwards RR	6	3/3	Mean: 22 ± 4^SD^	Nx	B: 6 mg/kg	i.m.	-
[17] Julien N	20	10/10	Female: 31 ± 8; Male: 28 ± 8	Nx	B: 0.28 mg/kg	i.v.	-
[18] Robertson LJ	32	17/15	Median: 19; Range: 17–39	Nx	B: 80 microg/0.2 ml (burn site)	s.c.	-
[19] Rennefeld C	24	24/0	26 ± 5	Nx	B: 0.15 mg/kg + I: 0.2 mg/kg/h	i.v.	-
[20] Leonard G	21+3^¤^	12+1/9+2	25 ± 6	Nx	B: 0.14 mg/kg x 2; B: 0.02 mg/kg x 2	i.v.	-
[21] Sprenger C	20	20/0	Mean: 26 ± 1^SD^	Nx	B: 0.15 mg/kg + I: 0.2 mg/kg/h	i.v.	-
[22] King CD	33	16/16	Mean: 24 ± 4^SD^	NTx	50 mg	p.o.	-
Repetitive Transcranial Magnetic Stimulation Models							
[23] de Andrade DC	36	24/12	Mean: 29 ± 6^SD^	Nx	B: 0.1 mg/kg + I: 0.1 mg/kg/h	i.v.	-
[24] Taylor JJ	24	12/12	Mean: 25 ± 3^SD^	Nx	B: 0.1 mg/kg	i.v.	Capsaicin topical
[25] Taylor JJ	14	NR	Range: 18–45	Nx	B: 0.1 mg/kg	i.v.	Capsaicin topical

^§^ not interfering with the MOR-antagonist assessments (drugs without administration route stated are i.v.).

^¤^ 3 additional volunteers were included due to unintended ʻcarry-overʼ (sequence) effects.

^#^ 12 volunteers total (2 volunteers did not participate in the naloxone parts of the study).

^SD^ standard deviation.

^**A**^ = age presented separately for each of the 6 groups of volunteers

**B** = bolus (up to 4 min administration time allowed); **F** = female; **I** = infusion; **M** = male; **ITP** = iontophoresis; **N.R.** = not reported; **NTx** = naltrexone; **Nx** = naloxone; **SD** = standard deviation; **TCI** = target-controlled infusion (total dose indicated).

#### 3.6.2 ʻSensitizingʼ Test Paradigms

Demographics are presented in Table 4. The total number of subjects in the STP-studies was 1,048, with a median (IQR) number in each study of 14.5 (11.3 to 23.8) subjects. The second largest (n = 158) [60] and the third largest (n = 151) [61] study reported partially duplicate data [61]. One study [41] was a companion study to a previously published study [85]. Two studies did not report the gender of the subjects [43,48], but based on calculations from the remaining 36 studies, the gender ratio (males/females) was 1.4 (601/430). Only 8 studies rendered information concerning body weight [31,32,34,36,49,51,53] or BMI [56], a detail of some importance, since 9 of the studies used weight-based infusion regimens [28–32,36,49,55,57]. Eight of the studies included patients with fibromyalgia [33,40], chronic low back pain [41,42], borderline arterial hypertension [49], bulimia nervosa [51] or major depression [52], but these data are not presented in the present review.

**Table 4:** ʻSensitizingʼ Test Paradigms: Demographics and Drugs.

[Ref.] First Author	N	Male/Female	Age (yr)	Drug	Dose	Administration	Additional drugs^§^
ʻSENSITIZINGʼ TEST PARADIGMS							
Secondary Hyperalgesia Models							
[26] Mikkelsen S	23	23/0	NR	Nx	B: 0.8 mg/15 min + 0.4 mg/h	i.v.	Ketamine
[27] Brennum J	24	24/0	24; Range: 20–31	Nx	B: 0.4 mg; B: 10 mg	i.v.	-
[28] Koppert W	13	13/0	31 ± 5	Nx	B: 10 microg/kg	i.v.	Remifentanil
[29] Koppert W	15	12/3	29 ± 6	Nx	B: 0.05, 0.5, and 5.0 microg/kg; TCI: 0.16, 1.6 and 16 microg/kg	i.v.	-
[30] Chu FL	9	9/0	30 ± 9	Nx	B: 0.1 mg/kg	i.v.	Remifentanil
[31] Pereira MP	22	11/1	F: 23 ± 1; M: 25 ± 2	Nx	B: 21 microg/kg	i.v.	-
Summation Models							
[32] Benedetti F	173	90/83	A	Nx	B: 0.14 mg/kg	i.v.	Capsaicin injection
[33] Price DD	14^A^	0/14	Mean: 46	Nx	B: 0.8 mg	i.v.	Fentanyl
ʻPainʼ Models							
*Capsaicin*:							
[34] Graven-Nielsen	15	15/0	Mean: 24; Range: 21–31	Nx	I: 0.8 mg /15 min + 0.5 mg /75 min	i.v.	Capsaicin injection
*Capsaicin & heat*:							
[35] Drummond PD	14	7/7	Mean: 22 ± 6^SD^	Nx	ITP: 0.5 mM	ITP	Capsaicin topical
[36] Anderson WS	9	5/4	Mean: 29 ± 5^SD^	Nx	B: 0.1 mg/kg	i.v.	Capsaicin topical
*Comb*. *modalities*, *others*:							
[37] Grevert P	30	15/15	NR	Nx	B: 1 mg/2 mg^#^; B: 10 mg	i.v.	-
[38] McCubbin JA	16	16/0	Range: 18–24	Nx	I: 8 mg	i.v.	-
[39] Stacher G	24	12/12	Range: 19–33	Nx	I: 5 mg; I: 20 mg	i.v.	-
[40] Younger JW	10^B^	0/10	Mean: 55 ± 8^SD^	NTx	50 mg	p.o.	-
[41] Bruehl S	39^C^	11/28	Mean: 31 ± 8^SD^	Nx	I: 8 mg	i.v.	-
[42] Bruehl S	31^D^	13/18	Mean: 34 ± 10^SD^	Nx	I: 8 mg	i.v.	Morphine
*Electrical*:							
[43] El-Sobky A	5	NR	NR	Nx	I: 0.4 mg; I: 0.8 mg	i.v.	-
[44] Buchsbaum MS	21	10/11	Mean: 20	Nx	I: 2 mg	i.v.	-
[45] Bromm B	15	15/0	Range: 21–29	Nx	32 mg^E^	p.o.	Tilidine
*Ischemia*							
[46] Grevert P	12	6/6	Median: 28	Nx	B: 10 mg; B: 2 mg	i.v.	-
[47] Grevert P	12	12/0	Mean: 25 ± 3^SD^	Nx	B: 10mg + I: 6 mg/h (8 hr); B: 2 mg + I: 1.2 mg/h (8 hr)	i.v.	-
[48] Posner J	12	NR	Range: 20–46	Nx	I: 2 mg^F^	i.v.	Codeine p.o.
*Mechanical*:							
[49] Schobel HP	9^G^	9/0	Mean: 25 ± 6^SD^	Nx	I: 0.15 mg/kg	i.v.	-
[50] Cook DB	12	12/0	Mean: 24 ± 4^SD^	NTx	50 mg^H^	p.o.	Codeine p.o.
*Thermal*:							
[51] Lautenbacher S	11^I^	0/11	Mean: 23 ± 3^SD^	Nx	I: 5 mg	i.v.	-
[52] Lautenbacher S	10^J^	12/8	Mean: 36 ± 11	Nx	I: 5 mg	i.v.	-
[53] Al’Absi M	26	15/11	Mean: 21 ± 9	NTx	50 mg	p.o.	
[54] Borras MC	10	10/0	Mean: 32 ± 7	Nx	B: 4 mg	i.v.	-
[55] Kern D	12	6/6	Range: 21–38	Nx	B: 0.1 mg/kg; I: 0.1 mg/kg/h (0.05 mg/kg)^K^	i.v.	Ketamine
[56] Kotlyar M	19	9/10	Mean: 26 ± 7	NTx	50 mg	p.o.	-
[57] Schoell ED	16^L^	8/8	Mean: 29 ± 5	Nx	B: 0.15 mg/kg; I: 0.2 mg/kg/h	i.v.	-
[58] Pickering G	10^M^	10/0	Mean: 26 ± 2^SD^	Nx	I: 8 mg^N^	i.v.	Paracetamol i.v.
Nociceptive Reflex Models							
[59] Boreau F	10	6/4	Range: 22–33	Nx	B: 0.8 mg	i.v.	-
[60] France CR	158	85/73	Mean: 19 ± 2^SD^	NTx	B: 50 mg	p.o.	-
[61] France CR	151	83/68	Mean: 19 ± 2 ^SD^	NTx	B: 50 mg	p.o.	-
Miscellaneous Models							
[62] Eissenberg T	12	8/4	Mean: 22 ± 3^SD^	NTx	B: 50 mg	p.o.	Oxycodone
[63] Robertson LJ	24	9/15	Median: 26; Range: 17–39	Nx	B: 80 microg/0.2 ml (burn site)	s.c.	Fentanyl
**Total all studies**	**1,477**						

^§^ not interfering with the MOR-antagonist assessments (drugs without administration route stated are i.v.).

^#^ 1mg: cold water challenge; 2mg: ischemic pain challenge.

^A^ study includes fibromyalgia patients (n = 15, data not reported here).

^B^ study includes fibromyalgia patients (n = 10, data not reported here).

^C^ study included patients with chronic low back pain (n = 37; data not reported here) and 2 healthy subjects on antidepressant medication.

^D^ study includes chronic low back pain patients (n = 45, data not reported here).

^E^ study includes treatment arms of combinations of tilidine (100 mg) and naloxone (8–32 mg; data not reported here).

^F^ study includes treatment arms with codeine (60 mg p.o.) and codeine/naloxone (2 mg i.v.; data not reported here).

^G^ study includes subjects with borderline hypertension (n = 21, data not reported here).

^H^ study includes treatment arm with codeine (60 mg p.o.; data not reported here).

^I^ study includes patients with bulimia nervosa (n = 10) and anorexia nervosa (n = 10; data not reported here).

^J^ study includes patients with major depression (n = 20; data not reported here).

^K^ study includes placebo-controlled treatment arm with ketamine (0.4 mg/kg; data not reported here).

^L^ the total number of subjects were 20 (4 were excluded).

^M^ the total number of subjects were 12 (2 were excluded).

^N^ study includes treatment arms with paracetamol (1g i.v.) and paracetamol/naloxone (8 mg i.v.; data not reported here).

^SD^ standard deviation.

For explanation of abbreviations, please, refer to legend Table 3.

### 3.7 MOR-antagonists

#### 3.7.1 ʻInhibitoryʼ Test Paradigms

Naloxone was used in 24 [1–21,23–25] studies and naltrexone in 1 study [22] (Table 3). Naloxone was administered IV in 21 studies [1–10,12–15,17,19–21,23–25], IM in 2 studies [11,16] and SC in 1 study [18]. In the naloxone studies, estimated from a mean body-weight of the subjects of 70 kg [31] (Table 3), the IV-doses ranged between 6 to 350 microg/kg [14,19] and the IM-doses between 17 to 6,000 microg/kg [11,16]. The estimated weighted mean dose of parenterally administered naloxone was 195 microg/kg. One study used two-doses of naloxone [20]. Naltrexone was administered PO in a dose of 0.71 mg/kg [22]. In all studies normal saline was used as placebo tested against MOR-antagonists.

#### 3.7.2 ʻSensitizingʼ Test Paradigms

Naloxone was used in 31 studies [26–39,41–49,51,52,54,55,57–59,63] and naltrexone in 7 studies [40,50,53,56,60–62] (Table 4). Naloxone was administered IV in 28 studies [26–34,36–39,41–44,46–49,51,52,54,55,57–59], SC in 1 study [63], PO in 1 study [45] and by iontophoresis in 1 study [35]. In the naloxone studies, estimated from a mean body-weight of the subjects of 70 kg [31] (Table 4), the IV-doses ranged between 6 to 827 microg/kg [27,43,47] and the PO-dose was 457 microg/kg [45]. In one dose-response study target-controlled infusion of naloxone was used [29] in doses ranging from 0.21 to 21 microg/kg. The SC-dose, 1 microg/kg, was minute and only intended for a local effect. The estimated weighted mean dose of IV administered naloxone was 125 microg/kg. Two separate doses of naloxone were used in 6 studies [27,37,39,43,46,47]. One study used a 3-dosing target-controlled infusion regimen [29]. Naltrexone was exclusively administered PO in doses of 0.71 mg/kg [40,50,53,56,60–62]. In all studies normal saline was used as placebo tested across the MOR-antagonists.

### 3.8 Adjuvant Drugs

#### 3.8.1 ʻInhibitoryʼ Test Paradigms

Adjuvant drugs were used in 4 studies either due to anxiolytic action (diazepam) [10,12] or to promote induction of pain (capsaicin [Table 3]) [24,25].

#### 3.8.2 ʻSensitizingʼ Test Paradigms

Adjuvant drugs were used in 11 studies due to the anti-hyperalgesic actions (codeine, fentanyl, ketamine, morphine, paracetamol, oxycodone, remifentanil, tilidine) [26,28,33,42,45,48,50,55,58,62,63] in 4 studies due to the pain-induction ability (capsaicin) [32,34–36] and in 2 studies due to development of opioid-induced hyperalgesia (remifentanil [Table 4]) [28,30].

### 3.9 Primary Test Stimuli

#### 3.9.1 Electrical Stimuli

ʻ*Inhibitory*ʼ *Test Paradigms*. Fourteen studies [1–8,10–15] used electrical stimuli as primary test stimuli (Table 5): 9 studies [1,2,5,7,8,10,12,14,15] used transcutaneous stimulation, while 4 studies [3,4,11,13] used non-invasive dental (pulpal) stimulation. Sural nerve-stimulation was used in 6 studies [1,2,10,12,14,15], tibial nerve-stimulation in 2 studies [1,12], alveolar nerve-stimulation in 4 studies [3,4,11,13] and supraorbital nerve-stimulation in 1 study [5]. In 6 studies [1,2,10,12,14,15] the nociceptive flexion reflex (NFR; also termed nociceptive polysynaptic reflex [NPR]) was elicited by sural nerve-stimulation and EMG-recordings of the RIII component from the biceps femoris muscle or the rectus femoris [15]. In 2 of these studies [1,12] the monosynaptic spinal reflex (MSR) was elicited by tibial nerve-stimulation and the EMG-recording of the H-component from the soleus muscle. A detailed description of the characteristics of the electrical stimuli is presented in Table 5.

**Table 5:** ʻInhibitoryʼ Test Paradigms: Testing Methods and Results.

[Ref.] First Author	Primary Test Stimuli	Conditioning Stimuli	Outcome Variables^△^	Main Findings
ʻINHIBITORYʼ TEST PARADIGMS				
Conditioned Modulation Models				
[1] Willer JC	MSR (Tibial-TNS + EMG H-S); NPR (Sural-TNSA + EMG RIII-BF)	NS: Sural noxious TNS; CAS: Warning announcement + randomized tactile /noxious stimuli	Reflex amplitudes (MSR [H]), reflex thresholds (NPR [RIII]), HR and RR	Nx facilitated the MSR (H-reflex), decreased the NPR (RIII) threshold and increased magnitude of autonomic variables, in response to noxious sural nerve conditioning stimulation
[2] Willer JC	NPR (Sural-TNSA + EMG RIII-BF)	NS: Sural noxious/tactile TNS; CAS: Warning announcement + randomized tactile /noxious stimuli	Reflex thresholds (NPR [RIII]), HR and RR	Nx reversed the increase in NPR (RIII) threshold responses to repetitive stress stimuli
[3] Pertovaara A	DEPTA	HF-TENSA	DEPT	Nx did not affect increases in DEPT induced by HF-TENS
[4] Pertovaara A	DEPTA; Thermal thresholds (TTA)	Arm ischemia (SETT)	VAS, heat thresholds, cold thresholds, electrical pain thresholds	Nx did not reverse ischemia induced elevation in dental electrical pain threshold but likely reduced the increase in heat thresholds (very low-powered study!)
[5] Willer JC	Blink reflex (BR-TNS)	HF-TNS	Nociceptive EMG- component of BR (R2)	Nx had no effect on the depression on the nociceptive blink reflex induced by high frequency non-noxious conditioning stimulation
[6] Pertovaara A	DEPTA	LF-TENS	EPT	Nx had no effect on the elevation of dental pain threshold due to non-noxious TENS.
[7] Buchsbaum MS	RESA	RES	CPS, EP	Nx increased pain sensitivity (enhanced amplitudes of EPs) after prolonged RES and attenuated RES-induced SIA
[8] Jungkunz G	FEPT	NS: FES; UCAS: Arithmetic stress (n = 15) or CWITA (0°C; n = 14)	EPT	Nx reversed the increases in electrical pain thresholds induced by CWIT.
[9] Janal M	HGSD_50%, 20_; CPTT_180s_; Radiant heat stimulation (RHSD)	Exercise (running 85% of MAC); Arm ischemia (SETT)	CPS, TTTo, WDL, HPR, psychometrics, endocrine response	Nx attenuated exercise induced ischemic but not thermal hypoalgesic effects. CPTT-data failed to demonstrate post-exercise hypoalgesia
[10] Willer JC	NFR (Sural-TNSA + EMG RIII-BF)	NS: Sural noxious/tactile TNS; CAS: Warning announcement + randomized tactile /noxious stimuli	VAS, reflex-thresholds (NFR [RIII])	Nx reversed the analgesic response and the increase in nociceptive reflex thresholds to repetitive stress stimuli, an effect mitigated by diazepam
[11] Ernst M	DEPTB	RDEPTB	EPT; Electrical discomfort thresholds	Nx had no effect on increase in dental electrical pain or discomfort thresholds induced by repetitive stimulation.
[12] Willer JC	NFR (Sural-TNSA + EMG RIII-BF); MSR (Tibial-TNS + EMG H-S)	NS: Sural noxious/tactile TNS; CAS: Warning signal + randomized tactile/noxious stimuli	VAS, reflex-thresholds (NFR [RIII]), HR, RR	Nx reversed the analgesic response, the increase in reflex thresholds and the increase in magnitude of autonomic responses to repetitive stress stimuli
[13] Olausson B	DEPTC	LF-TNS	EPT	Nx paradoxically prolonged the LF-TNS induced increase in EPT.
[14] Willer JC	NFR (Sural-TNSA + EMG RIII-BF)	HWIT (46°C)	Reflex-thresholds (NFR [RIII])	Nx completely blocked the inhibitory effect of DNIC on the nociceptive flexion reflex
[15] Poulsen L	NFR (Sural-TNSA + EMG RIII-RF)	CWIT (0.9°C)	NFR [RIII-RMS]), E-VAS	Nx near-significantly blocked the inhibitory effect of DNIC on the nociceptive flexion reflex and increased CPTT-induced pain, in extensive metabolizers of sparteine.
[16] Edwards RR	Thermal stimulations (TSHSA, HPT)	CWITA (1–3°C, repeated 4 times, duration not stated)^¤^	NRS, HPT, ABP,	Nx had no effect on DNIC-induced changes on heat pain perception, but seemed to increase cardiovascular reactivity to noxious cold
[17] Julien N	CWIT (12°C)	CWITC (12°C)	VAS	Nx inhibited the endogenous pain inhibitory systems activated by the spatial summation model
[18] Robertson LJ	HPT (RHSHG)	BIA + CWITA (2°C, repeated 6–10 times with 20 s interval)	HPT; VRS immersion	Locally administered Nx augmented sensitivity to cold water immersion tests (pain threshold, tolerance, rating). Locally administered Nx had modifying effects on heat sensitivity in non-burn skin after repeated cold water immersions
[19] Rennefeld C	HPT, HPR (TSHSB-stimuli); PDT (monofilaments 0.08–2,492 mN)	Repeated TSHSB (8 days)	HPT, HPR, PDT, VAS; (Day 1 + 8)	Nx had no effect on the magnitude of habituation for any of the stimulation sites (arm_stim_, arm_non-stim_ and leg).
[20] Leonard G	HSA	HF-TENSB	HPT, HPTo, HPR (COVAS)	High-dose Nx (0.28 mg/kg) blocked the analgesic effect of high-frequency TENS
[21] Sprenger C	HSB	CWITA (0°C; control 25°C)	EVAS, HPR, CPR, BOLD-responses	Nx compared to placebo:* increased pain ratings during CWIT; * did not alter pain ratings during phasic heat stimulation; * impaired the correlation between cold pain and endogenous analgesia; * reversed the coupling between ACC and DPCS
[22] King CD	HSC	CWITB (mean temperature 12.9°C)	EVAS, HPR, CPR; Psychometrics (CASE, PCS, SSE)	NTx abolished CPM induced decreases in HPR in subjects with low PCS-scores, but not in subjects with high PCS-scores
Repetitive Transcranial Magnetic Stimulation Models				
[23] de Andrade DC	CC	rTMS	CPT, CPRR	Nx attenuated the analgesic effect of M1-targeted repetitive transcranial magnetic stimulation (rTMS), but did not affect stimulation of DLPFC/PMC or sham controls.
[24] Taylor JJ	TTA, HSD	rTMS + Capsaicin (0.1%, topical, skin)	NRS, HPR; WDT, CDT, HPT, CPT, HPTo, CPTo; ± DLPFC-rTMS	Nx attenuated the analgesic effect of DLPFC-targeted repetitive transcranial magnetic stimulation
[25] Taylor JJ	TTA, HSD	rTMS + Capsaicin (0.1%, topical, skin)	NRS, HPR; HPTo; ± DLPFC-rTMS	Nx attenuated rTMS-induced analgesia, as well as rTMS-induced attenuation of BOLD signal response to heat-capsaicin stimuli throughout pain processing regions, including midbrain and medulla.

^△^ Outcome variable related to specific objectives of the review.

^¤^ Sequence I-II: temporal heat summation (forearm); sequence III-IV: HPT (forearm); sequences separated by 2 min.

**ABP** = arterial blood pressure; **ACC** = subgenual anterior cortex cinguli; **BIA** = burn injury A (probe area 0.8 cm^2^, 48°C, 2 min, application force 1N, arms/hands); **BDI** = Beck Depression Inventory; **BIB** = burn injury B (12.5 cm^2^, 47°C, 7 min); **BOLD** = blood-oxygen-level dependent contrast imaging; **BTS** = brief thermal sensitization (45°C, 3 min); **BR-TNS** = nociceptive component of blink reflex (supraorbital transcutaneous nerve stimulation, 0.1 ms duration, 0.15 Hz, 9–12 mA) assessed by integrated and rectified m. orbicularis oculi EMG [25–45 ms gated = R2 response]); **BS** = brush stimulation (1 cm stroke, 1 Hz, duration 25s, ISI 30s); **CAS** = conditioned aversive stimuli; **CASE** = cognitive affective side effects; **CC** = contact cold (30 x 30 mm^2^/ 25 x 50 mm^2^; -0.5°C/s or NR); **CDT** = cool detection threshold; **CEVAS** = continuous (0.2 Hz) EVAS; **CHA** = contact heat (30 x 30 mm^2^, 0.5°C/s); **CHB** = contact heat (2 cm^2^, 0.5°C/s); **CHC** = contact heat (3 x 3 cm^2^, 1°C/s); **CHEP** = contact heat evoked potentials (SSEP); **COVAS** = computerized visual analog scale; **CPR** = cold pain rating; **CPRR** = cold pain ratings at 5, 10 and 15°C, applied in a random order for 2 seconds; **CPS** = categorical pain scale; **CPT** = cold pain threshold; **CPTo** = cold pain threshold; **CPTT** = cold pressor test (ice-water); **CWIT** = cold-water immersion test (max. duration 2 min, hand); **CWITA** = cold-water immersion test A (approx. 7 min duration, hand/foot/leg); **CWITB** = cold-water immersion test B (40s, repeated 5 times, intersession resting period 3 min, fixed temperature level [8–16°C] corresponding to a CPR [“mild-to-moderate pain”: EVAS mean 42 (0–100)], foot); **CWITC** = cold-water immersion test C (8 consecutive immersions, 2 min duration, inter-stimulus interval 5 min, fingertip to shoulder and vice versa); **CWITD** = cold-water immersion test D (immersion 5 min duration, 10°C, hand, n = 18)); **DEPTA** = dental electrical pain threshold A (10 ms stimuli, 5 Hz); **DEPTB** = dental electrical pain threshold B (100 pulses, single pulse duration 0.1 ms, 0.6 s train, 0–0.5 mA); **DEPTC** = dental electrical pain threshold C (duration 22 ms, 6.2 Hz, 0–0.1 mA); **DLPFC** = left dorsolateral prefrontal cortex; **DPCS** = descending pain control system; **EDT** = electrical detection threshold; **EMG RII-BF** = electromyographic reflex responses [RII, latency 50–70 ms] from biceps femoris muscle [BF]; **EMG RIII-BF** = electromyographic reflex responses [RIII, latency 90–150 ms] from biceps femoris muscle [BF]; **EMG H-reflex** = electromyographic reflex responses [H] from soleus muscle [S]; **EP** = [somatosensory] evoked potentials; **EPT** = electrical pain threshold; **EPTo** = electrical pain tolerance; **EVAS** = electronic VAS; **FEPT** = finger electrical pain threshold (100 Hz, pulse-trains of 100 ms, duration 1s, 0–1.9 mA); **FES** = finger electrical stimulation (see FEPT); **fMRI** = functional magnetic resonance imaging; **FPP =** finger pressure pain (2,000 g applied at dorsal surface of middle phalanx of index finger for 1 min); **HF-TENSA =** high-frequency transcutaneous nerve stimulation A (bi-phasic stimulus, duration 0.6 ms, 100 Hz, 45 mA, cheeks); **HF-TENSB =** high-frequency transcutaneous nerve stimulation B (duration 0.06 ms, 100 Hz, segmental, sural nerve); **HF-TNS** = high-frequency transcutaneous nerve stimulation (duration 0.2 ms, 100 Hz, 2 mA, segmental/heterosegmental cutaneous nerves); **HGD80% max** = handgrip dynamometer 80% maximum grip strength for 90s; **HGSD** = handgrip strength measured by dynamometry (isometric, repeated contractions; set to 50% maximum grip strength 20 contractions/ set to 50% maximum grip strength/30% of maximal voluntary contraction/12-kg load 20 times); **HPR** = heat pain ratings; **HPT** = heat pain threshold; **HPTo** = heat pain tolerance; **HR** = heart rate; **HSA** = tonic heat stimulus with contact thermode (1 cm^2^, fixed temperature level corresponding to HPR 50 [COVAS 0–100], duration 120 s); **HSB** = phasic heat stimulus with contact thermode (9 cm^2^, 47.5°C, duration 5 s, repeated 64 times, inter-stimulus interval 45 s); **HSC** = tonic heat stimulus with contact thermode (5.3 cm^2^, fixed temperature level [46–50°C] corresponding to a HPR [“mild-to-moderate pain”], duration 30 s repeated 5 times, intersession resting period 3 min); **HSD** = tonic heat stimulus with contact thermode (9 cm^2^, fixed temperature level corresponding to HPR 7 [NRS 0–10], duration 22 s); **HSE** = phasic and tonic heat stimuli with contact thermode (6 cm^2^; 0.7°C/s or 35 s at HPT); **HSF** = tonic heat stimuli with contact thermode (3 x 3 cm^2^, 4 stimuli, 46°C, 25s, ISI 30s); **HSG** = phasic heat stimuli with contact thermode (3 x 3 cm^2^, 4 randomized stimuli, 43–48°C, duration 5 s, ISI 62 s, 10°C/s, repeated 10 times); **HSH** = stimulation with short phasic contact stimuli (stimulus area 0.6 cm^2^, peak temperature 51.8°C, 6 stimuli, ISI 15 s); **HSI** = phasic heat stimulus with RHSHG-technique (45°C, 5 s); **HWIT** = hot water immersion test (2 min, hand); **IDES** = intradermal electrical stimulation (rectangular, 0.5 ms duration, 2 Hz, high density); **ISI** = interstimulus interval; **IPT** = ischemia pain threshold; **IPTo** = ischemia pain tolerance; **ITC-SS** = infra-threshold cold stimulation—single stimuli (2.5 cm^2^, 0.2°C, 0.7 s stimuli, forearm); **ITC-TS** = infra-threshold cold stimulation—temporal summation (2.5 cm,^2^ 0.2°C, 0.7 s stimuli, train of 15 stimuli, 3 s inter-stimulus interval, hand); **LDPFC** = left dorsolateral prefrontal cortex; **LF-TENS** = transcutaneous electrical nerve stimulation (2.5 Hz, 0.2 ms duration, train of 5 impulses, 100 ms interval; stimulation areas 18.0 cm^2^); **LF-TNS** = low-frequency transcutaneous electrical nerve stimulation (duration 0.1 ms, 2 Hz, 9–45 V, 30 min stimulation hand/face [infraorbital nerve]); **MAC** = maximal aerobic capacity; **MPQ** = McGill Pain Questionnaire; **MSNA** = microneurographic recordings of sympathetic nerve activity to muscle; **MSR** = monosynaptic spinal reflex; **NFR** = nociceptive flexion reflex (same as NPR [RIII]); **N.R.** = not reported; **NRS** = pain ratings by numerical pain scale (0–10/100); **NS** = noxious stimuli; **NPR** = nociceptive polysynaptic reflex (same as NFR [RIII]); **PAA** = pressure algometry (1 cm^2^, 30 kPa/s); **PAB** = see PAA (1 cm^2^, 98 kPa/s); **PANAS** = Positive and Negative Affect Schedule; **PCS** = pain catastrophizing scale; **PDT** = pin-prick detection threshold; **PMES** = pain magnitude estimation scale; **PPR** = pin-prick pain rating; **PPT** = pin-prick pain threshold; **PRPT** = pressure pain thresholds; **PSR** = tactile polysynaptic reflexes (RII); **RDEPTB** = repetitive (1Hz, 3 min stimulation, inter-stimulus interval 7 min, 11 stimulation periods), DEPTB (see above); **RESA** = repetitive electrical skin stimulation (1–31 mA; other data NR); **RESB** = see RESA (1–31 mA, 1 mA increment, 93 stimuli, ISI 2.5 s); **RESC** = see RESA (200 Hz, duration 20 ms, ISI 20–40 s, repeated 40 times); **rTMS** = repetitive transcranial magnetic stimulation; **RHS** = radiant heat stimulation; **RHSD** = radiant heat stimulation by dolorimeter (0, 50, 340 and 390 mcal/s/cm^2^); **RHSHG** = radiant heat stimulation by halogen globe (95 mm^2^ [14] and [52]/ 28 mm^2^ [28] apertures, 0.5°C/s, maximum 45/48/49°C); **RMS** = root mean square; **RPS** = ratio proportional pain scale; **RR** = respiratory rate; **SBP** = systolic blood pressure; **SHA** = secondary hyperalgesia areas; **SETT** = modified submaximal effort tourniquet test; **SIA** = stress-induced analgesia; **SOWS** = subjective opioid withdrawal scale; **SP** = skin pinching (two opposed pegs, diameter 6 mm, force 18–25 N, stimulus duration 2 min, ISI 8 min, 5 anatomical sites); **SSE** = somatic side effects; **SSEP** = EP; **STCR** = supra-threshold cold stimulation pain ratings; **STAI** = State-trait Anxiety Inventory; **STHR** = supra-threshold heat stimulation pain ratings; **STH-SS** = supra-threshold heat stimulation—single stimulation (2.5 cm^2^, 52°C, duration 0.7 s/3.0 s, hand); **STH-TS** = supra-threshold heat stimulation—temporal summation (se **STH-SS** characteristics; train-of-ten); **STPPR** = suprathreshold pressure pain ratings (28 mm^2^, 700 kPa, 60 s); **Sural noxious/tactile TNS** = transdermal electrical sural nerve stimulation (randomized: 3 noxious stimuli [70–80 mA] or 2 tactile stimuli [4–8 mA]); **Sural-TNSA** = transdermal electrical sural nerve stimulation A (1 ms duration, train of 8–10, 20 ms train-duration [1]/1 ms duration, train-of-10, internal frequency 300 Hz, train-frequency 0.2 Hz, 10 mA [2]/ 1 ms duration, train-of-6, internal frequency 200 Hz, train-frequency 0.2 Hz, 10 mA [8]/ 1 ms duration, train-of-8, train-frequency 0.25 Hz, 0–30 mA [10]/ 1 ms duration, train-of-5, train-duration 20 ms, train-frequency 0.17 Hz [11]/ 1 ms duration, long lasting train (50–60 ms), internal frequency 300 Hz, 10 mA [48]; **Sural-TNSB** = transdermal electrical sural nerve stimulation B (300 Hz, 1 ms duration, long-lasting train, 5 mA); **Sural-TNSC** = transdermal electrical sural nerve stimulation C (volley of 5 stimuli, 1 ms duration, interstimulus interval 3 ms, 4–40 mA, 3 stimulation sessions spaced by 5 min); **TAM** = tibialis anterior muscle; **TCS** = thermal contact stimulators (3.1 cm^2^/0.8 cm^2^, 43/46/49°C, 2 x 6 stimuli, stimulus-duration max. 5 s, forearm); **TDES** = transdermal electrical stimulation (low density); **TESA** = transcutaneous electrical stimulation (duration 1 ms, 100 Hz, 0–6.4 mA, increments 0.05 mA, train-of-eight, randomized interval 15–25 s); **TESB** = see TESA (0.2 mA increments, ISI 1.2 s); **TG** = “thermal grill” is a device applied to the skin composed of six bars with alternating warm and cold temperatures (even- and odd-numbered) controlled by Peltier elements; **Tibial-TNS** = transdermal posterior tibial nerve stimulation (1 msec duration, 0.2 Hz); **TSHSA** = Temporal summation of heat stimuli A (49°C, stimulation area 9 cm^2^, stimulus duration 0.5 s, inter-stimulus interval 2.5 s, train of 10 stimuli, left forearm); **TSHSB** = Temporal summation of heat stimuli B (48°C, stimulation area 9 cm^2^, stimulus duration 6 s, inter-stimulus interval 4 s, train of 6 stimuli, 10 blocks separated by 40–60 s, left forearm); **TTA** = thermal thresholds (stimulation areas 1.8/9 cm^2^, lip/forearm); **TTB** = see TTA (6 cm^2^; 0.7°C/s, baseline either 32°C or 38°C); **TTTo** = tourniquet test tolerance; **VT** = vibratory threshold; **UCAS** = unconditioned aversive stimulus; **UV-burn** = ultraviolet “solar” stimulator (150W xenon lamp, UV_A_ [400 nm] and UV_B_ [290 nm], aperture 2 cm) skin exposure: 2.5 MED (“minimal erythemic dose”, arm); **VAS** = pain intensity and/or unpleasantness ratings by visual analog scale (0–100); **VNS** = visual numeric pain scale (0–100); **VRS** = verbal pain rating scale; **VS** = vibratory stimulation (0.8 cm^2^, 100 Hz, 0.5 s, displacement 1 mm); **VT** = vibratory threshold (stimulation area N.R., 3.7 N/cm^2^, 0.2 microm/s); **WCT** = warming by contact thermode (2 cm^2^, 37°C, > 80 min); **WDL** = withdrawal latency; **WDT** = warmth detection threshold.

ʻ*Sensitizing*ʼ *Test Paradigms*. Eight studies [32,39,43–45,59–61] used electrical stimuli as primary test stimuli (Table 6) and all used transcutaneous stimulation. Sural nerve-stimulation was used in 3 studies [59–61], and additional tibial nerve-stimulation in 1 study [59]. In the former studies the nociceptive flexion reflex was elicited by sural nerve-stimulation and EMG-recordings of the RIII component from the biceps femoris muscle [59–61]. In one of the studies [59] the monosynaptic spinal reflex was elicited by tibial nerve-stimulation and the EMG-recording of the H-component from the soleus muscle. In this study [59] tactile polysynaptic reflexes (PSR) were additionally elicited from sural nerve-stimulation and EMG-recordings of the RII-component from the biceps femoris muscle. A detailed description of the characteristics of the electrical stimuli is presented in Table 6.

**Table 6:** ʻSensitizingʼ Test Paradigms: Testing Methods and Results.

[Ref.] First Author	Primary Test Stimuli	Conditioning Stimuli	Outcome Variables^△^	Main Findings
ʻSENSITIZINGʼ TEST PARADIGMS				
Secondary Hyperalgesia Models				
[26] Mikkelsen S	Nylon monofilament (1,150mN) Brush	BIB	SHA, HPT	Nx no effect on SHA or HPT
[27] Brennum J	Nylon monofilament (1,150mN) Gauze swab	BIB	SHA, HPT, STHR, VS, STPPR, VAS	Nx no effect of on SHA or on other outcomes
[28] Koppert W	Nylon monofilament (450mN), Cotton-wool tip	IDES; TDES	SHA, NRS; Allodynic areas	Nx associated with trend in increase in SHA. Nx increased NRS during IDES. Nx no effect on allodynic areas
[29] Koppert W	Nylon monofilament (450mN), Cotton-wool tip	IDES	SHA; Allodynia areas; NRS	Nx (2 highest doses) increased SHA and NRS during IDES. Nx tended to revert the decrease of allodynic areas
[30] Chu FL	Non-flexible steel wire (160 mN)	IDES	SHA, VAS	Nx no effect on the SHA
[31] Pereira MP	Nylon monofilament (890mN)	BIB; BTS	SHA, HPT, WDT, PPT	Nx did not reinstate SHA after resolution of a burn injury. Nx no effect on SHA during BTS
Summation Models				
[32] Benedetti F	Capsaicin (10 microg, s.c.); TDES	Capsaicin (10 microg, s.c.)	Placebo response; NRS	Nx completely abolished the spatial-specific placebo response
[33] Price DD	STH-SS; ITC-TS	STH-TS; ITC-TS	VAS, EPT	Nx did not have an effect on the study variables compared to placebo.
ʻPainʼ Models				
*Capsaicin*				
[34] Graven-Nielsen	PAA, PPT (nylon monofilament, 1,237 mN)	Capsaicin (50 microg, i.m. in TAM)	EVAS, PRPT, PPT; (sensitivity at 6 sites on both legs)	Nx had no effect on pressure or pinprick pain thresholds during capsaicin-induced muscle pain.
*Capsaicin & heat*				
[35] Drummond PD	Radiant heat stimulation (RHSHG)	Capsaicin (0.6%, 400 microL skin)	HPT	Iontophoretically applied Nx and saline increased radiant heat sensitivity induced by capsaicin. After “body cooling” the Nx site was less sensitive to heat than the saline site
[36] Anderson WS	WCT	Capsaicin (10%, 35 mg, skin)	PMES	Nx significantly increased normalized pain ratings compared to placebo and baseline
*Comb*. *modalities*, *others*				
[37] Grevert P	HGSD_12 x 20_, CWITD	Arm ischemia (SETT)	NRS, psychometrics	Nx had no effect on pain induced by arm ischemia and cold-water immersion
[38] McCubbin JA	CPTT_90s_; HGD_80% max._	-	Pain rating (method N.R.); SBP	Nx did not affect pain ratings for cold pressor or handgrip challenge. Nx did not affect relationship between SBP and pain ratings
[39] Stacher G	TESA; RHS (method N.R.)	-	EPT, EPTo, HPT	Nx did not affect EPT or EPTo. Nx was associated with a slight but statistically significant increase in HPT
[40] Younger JW	CHA, CC, PAB	-	HPT, HPTo, CPT, CPTo, PRPT, SOWS	NTx did not affect thermal pain sensitivity or SOWS. NTx was associated with a slight but statistically significant increase in PRPT
[41] Bruehl S	FPP, HGSD_50%, 5 min_	-	IPT, IPTo, VAS, NRS, psychometrics (BDI, STAI, PANAS, MPQ)	Nx and placebo used as tools in estimating individual measures of endogenous opioid (EO) function
[42] Bruehl S	CHB, HGSD_50%, 8 min_	Arm ischemia (SETT)	IPT, IPTo, HPT, HPTo, VAS, NRS	Nx, morphine and placebo used as tools in estimating individual measures of endogenous opioid (EO) function
*Electrical*				
[43] El-Sobky A	TESB	-	EDT, EPT, EPTo	Nx did not affect electrical thresholds
[44] Buchsbaum MS	RESB	-	EP, CPS	Nx increased pain perception in pain insensitive individuals indicating a modulatory effect of endogenous opioid system. Nx did not affect the EP amplitude
[45] Bromm B	RESC	-	SSEP, CPS	Nx had no effect on pain perception but marginally increased SSEP amplitudes compared to placebo
*Ischemia*				
[46] Grevert P	HGSD_12 x 20_	Arm ischemia (SETT)	NRS, psychometrics	Nx had no effect on ischemic arm pain
[47] Grevert P	HGSD_12 x 20_	Arm ischemia (SETT)	VAS, psychometrics	Nx had no effect on ischemic arm pain
[48] Posner J	HGSD_50%_	Arm ischemia (SETT)	EVAS	Nx did not produce hyperalgesia or inhibited placebo analgesia
*Mechanical*				
[49] Schobel HP	SP	-	NRS, MSNA	Nx increased pain ratings and increased MSNA responses to pain
[50] Cook DB	HGSD_30%_	-	pain ratings (RPS), MSNA,	NTx has no effect on forearm musclepain, or MSNA during high-intensity handgrip tofatigue
*Thermal*				
[51] Lautenbacher S	HSE	-	HPT	Nx did not affect pain thresholds following phasic or tonic heat stimuli
[52] Lautenbacher S	TTB, VT	-	WDT, CDT, HPT, VT	Nx did not affect thermal or vibratory thresholds
[53] Al’Absi M	CHB, CPTT	-	HPT, HPTo, HPR (VNS), CPTT-rating (VNS)	NTx did not affect heat perception but was associated with reduced pain ratings during the CPTT
[54] Borras MC	BS, HSF	-	CEVAS, fMRI	Nx increased the “late” pain response after single heat stimuli. Nx produced activation of several brain regions enhanced by heat pain perception
[55] Kern D	TG	-	HPT, CPT, paradoxical pain*, STHR, STCR	Nx did not affect pain perception using the “thermo-grill illusion effect”
[56] Kotlyar M	CPTT	-	CPTT-rating (MPQ)	NTx did not affect pain perception during the CPTT
[57] Schoell ED	CHC, HSG	-	VAS, BOLD-signals,	Nx increased the intensity ratings for non-noxious heat stimuli. Nx affected the BOLD-signals in the ACC
[58] Pickering G	HSH	-	CHEP	Nx did not affect SSEP
Nociceptive Reflex Models				
[59] Boreau F	MSR (Tibial-TNS + EMG H-S); PSR (Sural-TNSB + EMG RII-BF); NPR (Sural-TNSA + EMG RIII-BF)	This study is not a DNIC study but uses stimulation techniques normally used in DNIC research	Reflex amplitudes (MSR [H]) and reflex thresholds (PSR [RII], NPR [RIII])	Nx facilitated the MSR (H-reflex), but did not affect the PSR (RII-reflex) or the NPR (RIII)
[60] France CR	NFR (Sural-TNSC + EMG RIII-BF); EPT (Sural-TNSC)	This study is not a DNIC study but uses stimulation techniques normally used in DNIC research	Reflex thresholds (NFR [RIII]), EPT, VRS	NTx did not affect the NFR threshold. NTx was associated with increased pain ratings during NRF-assessments in women. NTx was associated with increased EPT in men.
[61] France CR	NFR (Sural-TNSC + EMG RIII-BF); EPT (Sural-TNSC)	This study is not a DNIC study but uses stimulation techniques normally used in DNIC research	Reflex thresholds (NFR [RIII]), EPT, EPTo, VRS	NTx was associated with hypoalgesic responding in terms of decreased NFR-activity, lower EPT and EPTo.
Miscellaneous Models				
[62] Eissenberg T	TCS	UV-burn#	EVAS	NTRx reversed oxycodone induced antihyperalgesia in UV-exposed skin
[63] Robertson LJ	HSI (RHSHG); PPR (monofilament 121 mN)	BIA	EVAS; HPT, HPR, PPR	Locally administered Nx antagonizes local antihyperalgesic effects of fentanyl in a burn, in regard to HPT, HPR, PPR

^△^ Outcome variable related to specific objectives of the review.

^#^ short lasting erythema and heat hyperalgesia.

* pain produced by a combination of non-noxious warmth and cool.

For explanation of abbreviations, please refer to legend Table 5.

#### 3.9.2 Mechanical Stimuli

ʻ*Inhibitory*ʼ *Test Paradigms*. One study used pin-prick stimulations by nylon filaments [19] with bending forces from 0.08 mN to 2,492 mN [19] for assessments of detection thresholds (Table 5). One study used isometric handgrip strength measured by dynamometry [9].

ʻ*Sensitizing*ʼ *Test Paradigms*. Innocuous stimuli with brush [26], cotton-wool [28,29], or gauze swabs [27] were used for assessment of allodynia (Table 6). Pin-prick stimulations used in 8 studies, were by nylon filaments [26–29,31,34,63] with bending forces 121 mN to 1,237 mN [34,63], or a non-flexible steel wire [30], and, were used for assessments of secondary hyperalgesia areas [26–31], pain thresholds [31,34] and pain ratings [63]. Pressure algometry was used in 4 studies for assessments of pain thresholds [34,40] and suprathreshold pain ratings [27,41]. Handgrip strength was measured by dynamometry in 8 studies [37,38,41,42,46–48,50] and skin pinching in 1 study [49]. Vibratory stimulation was used in 1 study [27].

#### 3.9.3 Thermal Stimuli

ʻ*Inhibitory*ʼ *Test Paradigms*. Contact thermodes were used in 9 studies [4,16,19–25] with contact areas ranging from 1.0 cm^2^ to 9.0 cm^2^ [20,23] (Table 5). In 2 studies [9,18] radiant heat by a halogen globe directed at the skin, was used. Thermal thresholds were assessed in 7 studies [4,16,18–20,23,24] and thermal pain ratings in ten studies [9,16,17,19–25]. Eight studies used either phasic [16,19–21,23,24] or tonic heat stimuli [9,22,24,25], with temperatures ranging from 46°C to 50°C [22], stimulus duration of 5 [21] to 30 s [22] and repeated heat stimuli from 5 [22] to 64 times [21]. In 2 studies [16,19] temporal summation of heat stimuli in trains was used. In one of these [19] identical heat stimuli were used both as test stimulus and conditioning stimulus, in single mode and repeated mode, respectively. In 4 studies [9,15–17] cold-water immersion tests were used. In 1 study an ascending and descending spatial summation paradigm [17] was used, and in 2 studies [16,17] the cold-water immersion tests was employed both as test stimulus and conditioning stimulus. Two studies [24,25] used a block-testing technique, assessing heat allodynia with 3 separate 22 s stimuli using a temperature of the contact thermode calibrated at baseline corresponding to a pain rating of 7 (NRS, 0–10).

ʻ*Sensitizing*ʼ *Test Paradigms*. Contact thermodes were used in 15 studies [26,27,31,33,36,40,42,51–55,57,58,62] with the contact areas of the thermodes ranging from 2.0 cm^2^ to 12.5 cm^2^ [26,42] (Table 6). Three studies [35,39,63] used a source of radiant heat, in 2 of the studies by a halogen globe with apertures ranging from 94 mm^2^ to 1 cm^2^ [35,63]. Thermal thresholds were assessed in 12 studies [26,27,31,35,39,40,42,51–53,55,63] and thermal pain ratings in 7 studies [27,33,36,42,57,62,63]. Ten studies used phasic [33,39,42,51,53,57,62,63] and/or tonic [36,51,54] heat stimuli, with temperatures ranging from 43°C [57] to 51.8°C [58], stimulus duration of 0.7 s [33] to 35 s [51] and repeated up to 10 times [57]. In 1 study [33] identical heat and cold stimuli were used both as test stimulus and conditioning stimulus, in single mode and repeated mode, respectively. Cold-water immersion tests (0°C to 10°C) with pain ratings were used in 4 studies [37,38,53,56].

### 3.10 Conditioning Stimuli

#### 3.10.1 Electrical Stimuli

ʻ*Inhibitory*ʼ *Test Paradigms*. Six studies [1,2,7,10–12] used noxious electrical conditioning stimuli and 5 studies [3,5,6,13,20] used non-noxious electrical stimuli (peripheral conditioning). The studies used transcutaneous sural nerve-stimulation [1,2,10,12,20], transcutaneous nerve-stimulation [3,6,7,13], high-frequency (100 Hz) stimulation [3,5,20], or, low-frequency stimulation [13], or repetitive (1 Hz) dental stimulation [11]. Detailed stimulation characteristics are presented in Table 5.

ʻ*Sensitizing*ʼ *Test Paradigms*. Three studies used noxious electrical conditioning stimuli applied intradermally [28–30] or transdermally [28] (Table 6). Data from the 3 studies [59–61] using a classical electrical DNIC-paradigm are reported in paragraph 3.9.1, second subsection and Table 6.

#### 3.10.2 Mechanical Stimuli

ʻ*Inhibitory*ʼ *Test Paradigms*. Two studies [4,9] used the modified, ischemic submaximal effort tourniquet test, with assessment of hand grip strength, as conditioning stimulation (Table 5). One of these studies in addition used exercise (6.3 mile [10 km] run) at 85% of maximal aerobic capacity as a physiological conditioning stressor [9]. Another study employed 20 min leg and arm conditioning exercises on ergometers [13].

ʻ*Sensitizing*ʼ *Test Paradigms*. Six studies [37,41,42,46–48] used the modified, ischemic submaximal effort tourniquet test, with assessment of hand grip strength, as conditioning stimulation (Table 6).

#### 3.10.3 Thermal Stimuli

ʻ*Inhibitory*ʼ *Test Paradigms*. Nine studies [8,14–19,21,22] used thermal stimuli as a conditioning stimulus (Table 5). Cold-water-immersion tests (0 to 12.9°C) [8,15–18,21,22] and hot-water-immersion test (46°C) [14] were used in 8 studies. Two studies used cold-water-immersion tests (1 to 3°C) repeated 4 to 10 times [16,18]. Repeated heating, using a temporal summation pattern was used in 1 study [19].

ʻ*Sensitizing*ʼ *Test Paradigms*. Six studies [26,27,31,33,62,63] used thermal stimuli as a conditioning stimulus (Table 6). First degree burn injuries, leading to development of erythema and hyperalgesia, were induced by thermodes [26,27,31] (area 12.5 cm^2^, 47°C, 7 min), heat probes [63] (area 0.8 cm^2^, 48°C, 2 min) and UV-light [62] (150W xenon lamp, UV_A_ [400 nm] and UV_B_ [290 nm], aperture 2 cm), in 5 studies. Repeated suprathreshold heat stimuli and infrathreshold cold stimuli, respectively, attaining a temporal summation pattern, were employed in 1 study [33]. A brief conditioning sensitization heat stimulus (brief thermal sensitization) was used in 1 study [31].

#### 3.10.4 Pharmacological Stimuli

ʻ*Inhibitory*ʼ *Test Paradigms*. Conditioning with capsaicin 0.1% cream applied 30 min prior to heating with a contact thermode was used in 2 studies [24,25] (Table 5).

ʻ*Sensitizing*ʼ *Test Paradigms*. Conditioning procedures with capsaicin administered SC (10 microg) [32], IM (50 microg) [34], and, topically, in an aqueous solution (0.4 ml, 0.6%) [35] or cream (35 mg, 10%) [36], were used in 4 studies (Table 6). Two of these studies used heat to further sensitize the capsaicin treated areas [35,36].

#### 3.10.5 Miscellaneous Stimuli

ʻ*Inhibitory*ʼ *Test Paradigms*. In 3 studies [23–25] rTMS was applied as a conditioning analgesic stimulus. In the 2 latter studies [24,25] rTMS was targeted at the left dorsolateral prefrontal cortex (DLPFC) while in the third study [23] the analgesic effects were targeted at the right motor cortex (M1) and right DLPFC (Table 5).

### 3.11 Primary Objective and Outcome

Objective and outcome are related to the perspectives of this review and does not necessarily imply that these also are the main objectives and outcomes of the reviewed studies.

#### 3.11.1 ʻInhibitoryʼ Test Paradigms

*Conditioning Modulation Models*. The primary objectives of the conditioning modulation studies are schematically presented in Fig 2A (upper part) and summarized in Table 1. All studies used a paradigm where the reversal effect of MOR-antagonist across 5 apparently different conditioning models (as defined by the authors) was examined. The conditioning models included *stress-induced analgesia* (7 studies) [1,2,7–10,12], *spatial summation induced conditioning* (1 study) [17], *diffuse noxious inhibitory control* (DNIC; 4 studies) [4,14–16], *heterotopic noxious conditioning stimulation* (1 study) [21], *conditioned pain modulation* (CPM [heterotopic noxious conditioning stimulation and DNIC are by some authors^16;38^ included in the CPM-moiety; 1 study) [22], *repetitive noxious stimuli* (3 studies) [11,18,19] and *non-noxious frequency modulated peripheral conditioning* (5 studies) [3,5,6,13,20].

The outcome variables affected by the conditioning stimulation were pain perception [4,7,9,10,12,15–23,25], pain thresholds [3,4,6,8,11,13,16,18,19], nociceptive reflex thresholds (RIII) [1,2,10,12,14,15], autonomic parameters (heart rate, respiratory rate, arterial blood pressure) [1,2,12,16], sensory detection threshold [18], MRT (blood oxygen level dependent [BOLD])-responses [21], nociceptive component of blink-reflex (R2) [5], psychometrics [9,22] and SSEP [7]. The test stimuli were applied heterotopically [4–6,8,14–16,21], homotopically [1,2,7,10,11,17,18,20] or, both heterotopically and homotopically [3,9,12,13,19,22]. The outcomes of MOR-antagonist administration were reversal of the effects of the conditioning modulation (Fig 2), examining relative increases in pain perception, pain threshold, nociceptive reflex threshold or autonomic variables.

*Repetitive Transcranial Magnetic Stimulation Models*. The primary objective of the dorsolateral prefrontal cortex (DLPFC) targeted rTMS-studies was to examine the effect of a MOR-antagonist on stimulation-evoked analgesia [23–25] (Table 1). The test stimuli were applied homotopically, i.e., at the right side when rTMS was targeted at the left hemisphere and *vice versa*. The outcomes were changes in pain perception, pain threshold and pain tolerance (Table 5).

#### 3.11.2 ʻSensitizingʼ Test Paradigms

*Secondary Hyperalgesia Models*. The objectives were to examine if administration of a MOR-antagonist was associated with an increase in secondary hyperalgesia areas induced by a thermal injury [26,27,31], thermal suprathreshold stimulation [31] or noxious electrical high-intensity stimulation [28–30] (Table 2). Three studies examined either the effect of naloxone on ketamine-induced secondary hyperalgesia area [26] or opioid-induced hyperalgesia following remifentanil [28,30]. In both of these studies only data pertaining naloxone vs. placebo administration were considered relevant for this review. Another study examined re-instatement of secondary hyperalgesia area 72 hours after the burn-injury [31]. Outcomes were changes in secondary hyperalgesia areas, i.e. allodynia- or hyperalgesia-areas, evaluated by tactile or pin-prick stimuli, respectively (Table 6).

*Summation Models*. The objectives of the 2 summation studies were to examine the effect of a MOR-antagonist in regard to spatially directed expectation of pain [32], or temporal summation of phasic heat- and cold-stimuli [33] (Table 2). In the former, largest study (n = 173) of the review [32], SC injections of capsaicin were administered, unilaterally in the hand or in the foot, and expectations of analgesia were induced by application of a placebo cream, told to contain a ʻpowerful local anesthetic substanceʼ. This paradigm induced a placebo response in the treated body part, but not in untreated body parts. The objective of the study was to examine reversal effects of a MOR-antagonist on the placebo-response. In the latter study [33], repeated heat and cold stimuli, induced central sensitization, and the objective was to study the differential effect of naloxone on the first (single thermal stimuli [A-delta]) and second pain (repetitive thermal stimuli [C-fiber]) in the temporal summation process. The outcome parameters were in both studies pain ratings (Table 6).

*ʻPainʼ Models*. The objectives of these 25 studies were to examine the effect of MOR-antagonists on pain induced by capsaicin [34], capsaicin kindled by heat [35,36], cold [40,52,55], cold pressor test [37,38,53,56], electricity [39,43–45], heat [39,40,42,51–55,57,58], ischemia [37,42,46–48], or mechanical stimuli [37,38,41,42,46,47,49,50] (Table 2). The outcomes of MOR-antagonist administration compared to placebo, were changes in sensory thresholds [52], pain ratings [34,36–38,41,42,44–50,53–57], pain thresholds [34,35,39,40,42,43,51–53,55], pain tolerance [40–43,53], psychometrics [37,41,46,47], neuroimaging techniques [54,57], SSEP [44,45,58] or autonomic variables [38,49,50] (Table 6).

*Nociceptive Reflex Models*. The common objective of these 3 studies [59–61] was to examine the effect of MOR-antagonists on the thresholds of nociceptive flexion reflexes (Table 2). In addition, the first study [59] investigated non-nociceptive spinal reflexes, while the 2 other studies [60,61] investigated pain thresholds and pain ratings. The outcome parameters were nociceptive sural nerve stimulation induced changes in the biceps femoris muscle EMG-RIII component (Table 6).

*Miscellaneous*. The objective in the 2 studies was to examine the effects of MOR-antagonist on opioid induced antihyperalgesia following a burn-injury [62,63]. In both studies the MOR-antagonist was administered prior to opioid. The outcomes were pain ratings [62,63] and pain thresholds [63].

3.12 Secondary Objectives. Secondary objectives related to the administration of MOR-antagonists are presented in Tables 1 and 2.

### 3.13 Main Findings

#### 3.13.1 ʻInhibitoryʼ Test Paradigms

*Conditioned Modulation Models*. In the 7 SIA-studies [1,2,7–10,12], naloxone reversed the antinociceptive or analgesic effects of the conditioning stimuli, substantiated by decreases in threshold of the nociceptive flexion reflex (RIII) [1,2,10,12], threshold of the monosynaptic spinal reflex [1,12], electrical pain thresholds [8], and, increases in SSEP [7] and pain ratings (Table 5) [7,10,12]. One study demonstrated complete reversal of post-exercise *ischemic* hypoalgesia [9], although no response of naloxone to post-exercise *thermal* hypoalgesia was seen.

In the spatial summation induced conditioning study [17] naloxone reversed the spatial summation induced activation of the endogenous pain inhibitory system.

In 2 [14,16] of the 4 DNIC-studies [4,14–16], a naloxone-dependent complete reversal of the DNIC-induced increase in nociceptive flexion reflex [14] and an increased cardiovascular reactivity to tonic noxious cold stimulus [16] were demonstrated. However, in the latter study [16] no effect of naloxone on the heat pain perception (DNIC-efficiency [86]), compared to placebo, was observed. In the two remaining studies the findings were ambiguous [4,15]. In one of these studies [4] a significant naloxone-dependent effects were not demonstrated, though a likely reversal effect of naloxone on the conditioning-induced increase in heat pain thresholds was seen. In the other study [15] a trend (*P* = 0.07) towards a naloxone-dependent blocking effect of DNIC was observed and the authors attributed this to a type II error. The sample size in this cross-over study was 20 subjects (subgroup of extensive metabolizers of sparteine) indicating that the naloxone-dependent effect in this study, probably was rather weak.

In the heterotopic noxious conditioning stimulation study [21], naloxone generally did not affect pain ratings to phasic heat test stimuli during the cold-water immersion test (CWIT), however increased pain ratings to the tonic CWIT-conditioning stimulus were observed, compared with placebo treatment. The study also demonstrated an impaired correlation between the CWIT-pain ratings and the measure of endogenous analgesia (assessed by the phasic heat stimuli) in the naloxone-sessions compared to the placebo-sessions.

In the single CPM-study [22] (Table 5), it was demonstrated that naltrexone abolished a CPM-induced decrease in heat pain ratings, but only in subjects with low ratings on the pain catastrophizing scale (PCS).

The 3 studies [11,18,19] employing repetitive noxious stimulation, utilized very different methodological designs and reached different conclusions. One study observed that local administration of naloxone was associated with augmented sensitivity during repeated CWIT [18], while the other studies [11,19] were unable to demonstrate any naloxone-dependent effects on electrical pain thresholds [11] or on the magnitude of habituation in a complex model of repeated heat stimuli [19].

In 3 out of 5 studies [3,5,6,13,20] utilizing a non-noxious frequency modulated peripheral condition stimulation model, no effect of naloxone on the nociceptive component of the blink reflex [5] or dental electrical pain thresholds [3,6] was seen. In one study a paradoxically prolonged increase in the electrical dental pain threshold was observed after naloxone administration [13], indicating a hypoalgesic effect. A study using high-frequency transcutaneous electrical nerve stimulation induced thermal hypoalgesia was not affected by placebo or low-dose naloxone (0.04 mg/kg), but was blocked with high-dose naloxone (0.28 mg/kg) [20].

In summary, 8 studies [1,2,7–10,12,17], demonstrated consistently for SIA and for spatial summation induced conditioning, that conditioning effects were reversed by naloxone, corroborating a role of the endogenous opioid system in these testing models. For the remaining models [3–6,11,13–16,18–22] ambiguous findings on the MOR-antagonist efficacy in reversing the conditioning effects were reported.

*Repetitive Transcranial Magnetic Stimulation Models*. In these interesting sham-controlled studies with rTMS targeted at the dorsolateral prefrontal cortex (DLPFC) [23–25] and the motor cortex (M1) [23], pre-treatment with naloxone attenuated the analgesic effect indicating an endogenous opioid-dependent mechanism of rTMS (Table 6). Two of the studies [24,25] observed significant attenuating effects of naloxone on DLPFC-targeted rTMS, while the third study [23] only observed this for M1-targeted rTMS, but *not* for DLPFC-targeted rTMS.

#### 3.13.2 ʻSensitizingʼ Test Paradigms

*Secondary Hyperalgesia Models*. One study [29] demonstrated a dose-dependent naloxone response, with increasing magnitudes of secondary hyperalgesia areas induced by high intensity intradermal electrical stimulation. Interestingly, in 2 [28,29] of 3 studies [28–30] with intradermal electrical stimulation increased pain ratings, indicating heightened pain sensitivity, were observed following naloxone administration. However, in 5 [26–28,30,31] of the 6 secondary hyperalgesia area studies [26–31] no signs of naloxone-dependent increases in secondary hyperalgesia areas were observed (Table 6). However, one of the studies examined the *late* reinstatement of secondary hyperalgesia areas, 72 hrs after a mild burn-injury [31].

*Summation Models*. In the large placebo study [32] naloxone completely abolished the placebo response indicating that endogenous opioids, spatially modulate specific placebo responses. In the temporal summation study [33] using repeated phasic heat and cold stimuli, no effect of naloxone, compared to placebo, on thermal wind-up, “first” pain or “second” pain, was observed (Table 6).

*ʻPainʼ Models*. In the 18 studies [34,36–38,41,42,44–50,53–57] using pain ratings as primary outcomes, 6 studies [36,44,49,53,54,57] observed an MOR-antagonist dependent effect, while 10 studies did not observe any significant effect [34,37,38,45–48,50,55,56] (Table 6). Five studies, examining the response to MOR-antagonists, reported increases in ratings to electrical stimuli (only in pain-insensitive individuals) [44], non-noxious heat [57], noxious heat [36,54], and skin pinch [49], while 1 study reported paradoxically *decreased* pain ratings during the cold pressor test [53]. Thirteen studies did not report any MOR-antagonist dependent changes in pain ratings in regard to the cold pressor test [37,38,56], dynamometric tests [37,38,46,47,50], electrical stimuli [45], ischemia [41,42,48], phasic thermal stimuli [42,53,55], or, pressure and pinprick stimuli [34].

In regard to thermal thresholds 2 studies, assessing heat pain thresholds,[35,38] observed an *increase* in thresholds, following either local, iontophoretic [35] or systemic [38] administration of MOR-antagonist, indicating a hypoalgesic mechanism of action. However, 6 studies [40,42,51–53,55] could not substantiate any change in heat pain thresholds associated with administration of MOR-antagonist. Three of these studies [40,52,55], additionally, could not show any effects on cool detection, cold pain or cold tolerance thresholds. Two studies [39,43] examining electrical thresholds and 1 study examining vibratory thresholds [52] could not show any effect related to the systemic opioid-blockade. Three studies measured SSEP [44,45,58], but only in 1 study [45] MOR-antagonist dependent changes in SSEP were observed. In one [49] of 2 studies [49,50] using microneurographic recordings of sympathetic nerve activity (MSNA), an increase in activity related to the opioid-blockade was seen.

Interestingly, 2 neuroimaging studies [54,57], using fMRI [54] and the BOLD-contrast imaging technique [54,57] could substantiate functional changes, i.e., increases in activity of several brain regions [54] or blocked deactivation of the pregenual ACC (anterior cortex cinguli) [57], correlating with perceptual responses to noxious and non-noxious heat exposure, following administration of MOR-antagonist (Table 6).

Summarizing the results from the 25 studies, the direction of MOR-antagonist dependent effect on pain ratings, threshold assessments and SSEP appear quite ambiguous and inconsistent [42]. Any evidence for stimulation modality specific changes in response to MOR-antagonists is lacking. However, the results on heat stimuli from the 2 neuroimaging studies seem consistent and promising [54,57].

*Nociceptive Reflex Models*. In the first study [59] naloxone facilitated, i.e. increased the amplitude, of the monosynaptic spinal reflex, but did not affect the tactile polysynaptic reflex (RII). In 2 [59,60] of the 3 threshold studies [59–61] no effect of naloxone on the threshold of nociceptive flexion reflex (Table 6) could be demonstrated. In the third study [61] the results were somewhat at odds with previous findings, indicating lowered nociceptive flexion reflex activity (defined as the rectified biceps femoris EMG measured at the 90–150 ms post-stimulation interval) after administration of naltrexone: a hypoalgesic effect corroborated by the naltrexone-associated findings of significantly decreased pain ratings at electrical pain and tolerance thresholds. However, both in the second [60] and third study [61] administration of naltrexone during noxious sural nerve-stimulation was associated with increased pain in female subjects, while in male subjects naloxone administration was associated with an *increase* in electrical pain thresholds.

*Miscellaneous Models*. In the 2 burn-injury studies [62,63] a naloxone-dependent reversal of opioid-induced anti-hyperalgesia was demonstrated primarily by an increase in heat sensitivity (Table 6).

### 3.14 Adverse Effects, Withdrawals and Outliers

#### 3.14.1 ʻInhibitoryʼ Test Paradigms

Seven of the 24 studies described either drug-related adverse effects [1,22], withdrawals not related to administration of MOR-antagonist [13,21,24,25] or the occurrence of outliers [8]. In one study [22] adverse effects, termed “mild side effects”, like mental dulling, confusion, sedation and poor balance were reported. Unfortunately this study only described mean values of the adverse effects based on the group, while the absolute number of subjects experiencing the adverse effects was not given. In the study with the highest naloxone dosis, i.e. 6,000 microg/kg, the subjects were unable to tell the correct order (with a likelihood higher than chance) of active drug vs. placebo and no adverse effects were reported [16].

#### 3.14.2 ʻSensitizingʼ Test Paradigms

Thirteen of the 38 studies described either occurrence of drug-related adverse effects [26,27,38,39,42,51,62], withdrawals not related to administration of MOR-antagonist [30,36,38,53,57,58] or the occurrence of outliers [55]. Six subjects were reported to experience adverse effects: 1 subject had psychotropic effects due to ketamine [26], 1 subject developed a second degree burn injury [26] and 4 subjects experienced sensation of warmth, palpitations, drowsiness, nausea and vomiting [27,62]: in 3 of the subjects very likely related to administration of MOR-antagonist. In one study [39] the responses in the side effect questionnaires showed that tiredness, lightheadedness, nausea, abdominal “grumbling”, and mood changes were reported slightly more often after naloxone than after placebo. In another study [51] a "drowsiness" scale demonstrated higher values for naloxone than for placebo.

## Discussion

### 4.1 Main Results

The principal findings of this review on opioid-antagonism in experimental pain models, are *first*, that naloxone, in 10 out of 25 studies, utilizing an ʻinhibitoryʼ test paradigm, consistently reversed analgesic and anti-nociceptive effects of SIA [1,2,7–10,12] and repetitive transcranial magnetic stimulation [23–25], thus implicating a role of the endogenous opioid system. *Second*, in the remaining 15 out of 21 conditioned modulation studies, the effects of opioid-antagonism were negative (5 studies) [3,5,6,11,19], positive (5 studies) [13,14,17,18,20] or ambiguous (5 studies) [4,15,16,21,22]. *Third*, among the 38 studies utilizing a ʻsensitizingʼ test paradigm, in 5 [26–28,30,31] out of 6 studies examining secondary hyperalgesia models, an effect of MOR-antagonists could not be demonstrated. *Fourth*, only in 4 out of the 32 remaining ʻsensitizingʼ test paradigm studies, consistent effects of opioid-antagonism were demonstrated, i.e., in 2 neuroimaging studies [54,57] and 2 burn-injury studies assessing local hyperalgesic effect of the MOR-antagonist [62,63]. *Fifth*, and probably the most important finding, the plethora of stimulation modalities, conditioning methods, assessment methods and extent of opioid-blockade, preclude any attempt at quantitative evaluation of the retrieved studies.

### 4.2 Potential Clinical Implications

#### 4.2.1 ʻInhibitoryʼ Test Paradigm

The descending conditioned pain modulation system (DNIC or CPM; cf. 4.4.2) is considered an important factor regulating pain sensitivity in humans [87–89] and it has been suggested that pathological changes in the CPM-system are important for the development of chronic pain in chronic tension headache, fibromyalgia and persistent postoperative pain [33,88,90–94]. The CPM system is in part modulated by exogenous opioids: a sub-therapeutic dose of morphine may uncouple the conditioning system, deregulating the balance between pain sensitization and inhibition [14,95]. Impairment of the descending inhibitory systems, e.g., the EOS and CPM, may contribute to the trajectory from acute to chronic pain. Research in blockade of the opioid system may improve our understanding of the underlying pathophysiological mechanisms and may lead to a reformulation of strategies for the prevention and management of chronic pain.

#### 4.2.2 ʻSensitizingʼ Test Paradigm

Obviously, a number of scientific issues are common for the complementary test paradigms, ITP and STP, however, injury or disease related nociceptive input to the CNS may trigger a sustained excitability and increased synaptic efficiency in central neurons [93], i.e., central sensitization (CS), a stimulus-response enhancing mode which may contribute to the development and maintenance of a chronic pain state [93,96]. Animal data suggest that CS outlasts overt signs of hyperalgesia, in a silent form termed ʻlatent sensitizationʼ (LS). The LS far outlasts the conventional duration of the injury assessed by behavioral measures, but can be unmasked by administration of a centrally acting MOR-antagonist leading to “rekindling” or reinstatement of hyperalgesia [92,93,97]. Thus, post-injury pain remission is maintained in part by the EOS that masks the pro-nociceptive components of LS. ʻLatent sensitizationʼ could prime central nociceptive circuitry such that, when inhibitory systems fail, as upon exposure to excessive stress, a pain episode ensues [93,97]. The latent predisposition to relapse, may explain the episodic nature and vulnerability to stressors that accompany chronic pain states in humans [93,97].

### 4.3 Dose-issue

*First*, while the orally administered naltrexone dose was rather uniform 50 mg [22,40,50,53,56,60–62], the parenterally administered naloxone doses, ranged from 0.21 to 6,000 microg/kg [16,29]: a 29,000 fold difference in doses across studies (Tables 3 and 4)! This difference may obviously bias the study results, particularly considering the biphasic response pattern induced by MOR-antagonists (cf. 2.3). The dose-response issue, was examined by 8 studies [20,27,29,37,39,43,46,47], but only 2 studies recognized a dose-response pattern [20,29]. The ITP-study [20] comparing naloxone 0.04 mg/kg and 0.28 mg/kg, demonstrated a significant effect of the highest dose on blockade of the analgesic effect of high-frequency transcutaneous electrical nerve stimulation. However, there are some methodological objections to this study. The authors observed highly significant sequence effects: subjects randomized to receive placebo at their first session (naloxone administered at the second and third sessions) demonstrated a higher difference in pain scores compared to the naloxone sessions, than when placebo was administered at the third session (naloxone administered at the at the first and second sessions). This effect most likely was attributed to habituation generated by the conditioning electrical stimuli (cf. 4.4.1). Since only data from first sessions with placebo administration could be used, the authors in order to compensate for the unintended reduction in statistical power, in a *post-hoc* manner included 3 more subjects, most likely violating the study protocol. The STP-study [29] used successively, increasing naloxone doses of 0.21, 2.1 and 21.0 microg/kg, administered as target-controlled infusions. Significant dose-dependent increases in secondary hyperalgesia areas were demonstrated (in 2 out of 3 test sessions), and this study is the only, in a statistically correct way, to confirm a dose-response relationship for naloxone in the present review.

*Second*, the systemic bioavailability of orally administered naltrexone and naloxone are 5% to 60% [81] and, 2% to 3% [73], respectively. Thus, in studies using oral administration [22,40,45,50,53,56,60–62] a variable and low systemic bioavailability across subjects and studies may have influenced the results.

*Third*, the weighted mean dose of parenterally administered naloxone, was 158 microg/kg (11.0 mg for 70 kg BW). Human data, data based on a PET-study, demonstrated complete inhibition of [^11^C]-carfentanil binding to opioid-receptors following 100 microg/kg naloxone [98]. The authors are not aware of additional binding-studies and though the effective naloxone blocking dose may be lower, it is interesting that only in 19 out of 53 studies in the present review, with parenterally administered naloxone, a dose of ≥ 6 mg (86 microg/kg per 70 kg BW) was used. Considering the short half-life of naloxone it seems important to maintain steady effect compartment concentrations by target-controlled infusions.

### 4.4 ʻInhibitoryʼ Test Paradigms

#### 4.4.1 Stress-induced Analgesia

In the SIA-models two stimuli are required: an aversive stressor-stimulus and a noxious test-stimulus [99]. The aversive stimuli in the present SIA-studies [1,2,7,8,10,12], were intense, noxious electrical conditioning stimulation [1,2,7,10,12], (in 4 of the studies even termed ʻinescapableʼ shocks [1,2,10,12]), the cold pressor test [8], an arithmetic stressing test or a 6.3 mile run at 85% of maximal aerobic capacity [9] (Table 5). In the electrical studies an alternating procedure was used, randomizing sequences of high-intensity noxious and low-intensity tactile stimuli, in order to facilitate apprehension and discomfort, adding psychological factors to the test paradigm. Electrical noxious test-stimuli evaluating the conditioning effects were employed in all studies.

Transcutaneous electrical stimulation is a ubiquitous method used in pain research, due to its ease of use, flexibility, and in particular, due to its versatility, regarding stimulation rates, intensity adjustments and generation of sequence-patterns. The method elicits pain and hyperalgesia by direct axonal stimulation, bypassing the sensory nerve endings [100]. Electrical stimuli are thus probably more suitable for examination of central pain components, than ʻphysiologicalʼ thermal and mechanical stimuli, reflecting both peripheral and central components of the pain response [101]. High-intensity, noxious electrical stimulation (for stimulation characteristics cf. Table 5) is associated with activation of the EOS and an apparent stimulation-dependent decrease in pain ratings [28,29,102]. The degree of habituation, assessed as decrements in pain ratings, following 45–180 min continuous noxious electrical stimulation, is between 20–60%, using constant stimulation intensity [28,29,100,102,103]. Using a test paradigm, adjusting the current strength to a constant level of pain perception, the increase in stimulation intensity during 45 min of continuous stimulation, is 260% [103]! Thus, in the SIA-studies the decrements in pain ratings [7,10,12], increases in threshold of the nociceptive flexion reflex (RIII) [1,2,10,12], thresholds of the monosynaptic spinal reflex [1,12], electrical pain thresholds [8], and, decreases in SSEP [7], observed in controls, could in part be explained by habituation [7] (Table 5).

Prolonged and intense, noxious electrical conditioning stimulation activates a naloxone-sensitive (indicating recruitment of the EOS) and a naloxone-insensitive inhibitory system [29]. Interestingly, these findings are not modality specific, but also apply to noxious contact heat [104,105], laser stimuli [106] and capsaicin application [36].

In heat-models it has been shown, that habituation involves the descending antinociceptive system and in addition comprises a naloxone-insensitive component [19]. The fMRI-based studies demonstrated that part of the antinociceptive system, the rostral and subgenual anterior cingulate cortex (rACC/sgACC)) and the periaqueductal grey area, were involved in habituation, indicating a contribution of central pathways to the phenomenon of habituation [104,106].

Experimental factors that potentially may influence habituation and its central correlates are the stimulation modality, stimulation rate, the timeline of the stimulation, i.e. short-term or long-term habituation, and, the use of phasic or tonic stimulation patterns [19,103,104].

#### 4.4.2 Diffuse Noxious Inhibitory Control and Conditioned Pain Modulation

DNIC and CPM are synonymous terms, and it has recently been recommended that DNIC should be reserved for animal and CPM for human research [88]. In the present review the terms were used explicitly as stated in the studies.

The 4 DNIC-studies [4,14–16] presented similar findings in regard to the ʻclassicalʼ DNIC-paradigm: a high intensity noxious stimulus decreased the response or increased the threshold to a heterotopically applied pain-stimulus [87]. However, naloxone-induced reversal of the DNIC-effect was only unambiguously demonstrated in one of the studies [14], an effect that would have been anticipated since previous studies have indicated opioid-sensitive components of DNIC [14,87,107]. Among the 3 DNIC-studies [4,15,16] with ambiguous findings, two of the studies were low-powered [4,16] and the authors of one of the studies stated that the negative findings could likely be attributed to a lack of statistical power (n = 6) [16]. Interestingly, one of the studies [15], seemingly adequately powered, indicated a weak naloxone-dependent effect (cf. 3.13.1).

Although the CPM-study [22] partially demonstrated a naltrexone-dependent abolishment of the conditioning induced decrease in pain response (Table 5), the general ambiguous findings in the 5 studies [4,14–16,22] seem difficult to reconcile.

#### 4.4.3 Non-noxious Frequency Modulated Peripheral Condition Stimulation

The 5 studies on non-noxious frequency modulated peripheral conditioning did not show any consistent sign of involvement of the EOS [3,5,6,13,20].

#### 4.4.4 Repetitive Transcranial Magnetic Stimulation

The methodological qualities of these studies were among the highest in the present review. An advantage was that the studies used sham-controlled and placebo-controlled procedures. Two of the studies [24,25] observed significant attenuating effects of naloxone on DLPFC-targeted rTMS, while the third study [23] only observed this for M1-targeted rTMS. The studies utilized thermal test paradigms, although they differed in regard to pretreatment with capsaicin that was used in two of the studies [24,25], i.e., application of capsaicin may lead to more intense pain perception [108]. The studies also differed in regard to left [24,25] and right [23] hemisphere targeted stimulation.

### 4.5 ʻSensitizingʼ Test Paradigms

#### 4.5.1 Secondary Hyperalgesia Models

One study [29] demonstrated a dose-dependent naloxone response, with increasing magnitudes of secondary hyperalgesia areas induced by noxious, high intensity, intradermal electrical stimulation. Interestingly, in a preceding study by the same research group [28], using a nearly identical set-up, naloxone-infusion 10 microg/kg, was associated with a highly significant increase in secondary hyperalgesia area (140%) compared to pre-infusion values (*P* < 0.01). However, since the baseline values successively increased during the 30 min infusion period, the increase in secondary hyperalgesia areas compared to controls, did not reach significance (*P* = 0.16; deviations in baseline assessments in noxious electrical stimulation, cf. 4.4.1). The study abstract correctly indicated that naloxone resulted in increased pain ratings (ʻantianalgesiaʼ; *P* < 0.001), but, erroneously indicated that naloxone resulted in ʻmechanical hyperalgesia (*P* < 0.01)ʼ [28]. The third secondary hyperalgesia study using high intensity intradermal electrical stimulation, on remifentanil-induced opioid hyperalgesia (OIH) [30] was not able to demonstrate any effect of naloxone on secondary hyperalgesia. Though the study generally had a double-blind, controlled and randomized design, the naloxone part of the investigation did not include a placebo arm *per se*, but naloxone was administered blinded to the remifentanil and the placebo groups.

Consequently, in 5 of 6 secondary hyperalgesia studies obvious signs of EOS involvement could not be demonstrated, however, naloxone-dependent hyperalgesic responses during high intensity noxious electricity stimulation cannot be excluded. Recent data, examining latent sensitization [109] using a burn injury model [31] indicated that administration of a high dose of naloxone 2 mg/kg, 1 week after the injury, in 4 out of 12 subjects seem to be associated with late re-instatement of secondary hyperalgesia areas (submitted: Pereira MP et al. ʻEndogenous opioid-masked latent pain sensitization: studies from mouse to human.ʼ).

### 4.6 Study Bias

#### 4.6.1 General Issues

The bibliographic age and the exploratory nature of the studies should be taken into consideration when discussing study bias, i.e., the risk that the true intervention effect will be overestimated or underestimated (http://handbook.cochrane.org/ [accessed 07.24.2014]). The present review using the simple Oxford quality scoring system from 1996 [68] demonstrated a high likelihood of bias, due to inaccurate reporting of randomization and blinding procedures. The Oxford quality scoring system was chosen in respect of the bibliographic age of the studies since it presents a more lenient evaluation paradigm than more sophisticated methods like the Cochrane Collaboration’s tool for assessing risk of bias. Approximately 25% and 50% of the studies were published before 1984 and before 2000, respectively, while the early report on the Consolidated Standards of Reporting Trials (CONSORT) was published in 1996 [110], with revised versions 2001 [111] and 2010 [112]. Compliance to these standards has been a requirement for randomized controlled trials (RCTs) in a number of clinical journals for more than 17 years [110]. The CONSORT statement thus contains guidelines applicable to clinical RCTs, but is it relevant for experimental RCTs?

The editorial accompanying the first CONSORT publication stated: “It seems reasonable to hope that, in addition to improved reporting, the wide adoption of this new publication standard will improve the conduct of future research by increasing awareness of the requirements for a good trial. Such success might lead to similar initiatives for other types of research”[110]. Requirements for clinical RCTs as outlined in CONSORT and SPIRIT (Standard Protocol Items: Recommendations for Interventional Trials) [113], could also be considered essential for experimental research in order to heighten validity, reliability and reproducibility of data, facilitating accurate reporting, evaluation and interpretation of study-data. Guidelines for reporting animal research data, ARRIVE (Animals in Research: Reporting In Vivo Experiments) [114], based on the CONSORT criteria, have been published and even extrapolated for use in a review including human experimental research [115].

#### 4.6.2 Statistical Issues

The heterogeneity of the statistical methods used was considerable. Various ANOVA-methods, some rather advanced [24,42], were used in 41/63 studies, while *a priori* and *post-hoc* sample size estimations were only employed in 4/63 and 3/63 studies, respectively. Furthermore, in 49/63 studies, statistical methods aimed at reducing the risk of type I error (α [false positive]) associated with multiple comparisons, were not applied. It can readily be calculated that the likelihood by chance of achieving one or more false positive results, presenting as significant values (*P* < 0.05), during 5 and 10 uncorrected pair-wise comparisons, which was a normal procedure in the studies, are 23% and 40%, respectively, giving a high likelihood for falsely rejecting the null hypothesis [116,117]. A simple measure to attenuate the risk is to decrease the significance level to 1% which would give corresponding likelihoods of less than 4.9% and 9.6%, respectively. Conversely, the risk of committing a type II error (β [false negative]), i.e., indicating the power of the study, was generally not reported in the studies, although the limited sample size was discussed in 10/63 studies.

However, it could be argued that all the reviewed studies are experimental, and as such exploratory and hypothesis-generating in nature. In studies of healthy individuals, inferences from qualitative aspects, within-subject variances and fixed-effect models are often more important than inferences from quantitative aspects, between-subject variances and random-effect models, the latter being preferred in clinical research examining groups of patients [118]. The number of subjects needed is obviously much smaller for experimental research compared to clinical studies, mainly due to a much larger inherent biological variance in patients compared to healthy subjects. However, the exploratory nature of an experimental study is sometimes used as a an excuse for not adhering strictly to common statistical requirements [117]. For all research, decisions on the null hypothesis, primary and secondary outcomes, and, estimations of outcome variability (from pilot-data if not available from literature), minimal relevant differences, sample size and effect size calculations should be stated even in studies of exploratory nature. Otherwise, there is an obvious risk of wasting valuable research time and efforts, leading to ethical, economical or scientific dilemmas, which might impede future research [116,117,119].

#### 4.6.3 Methodological Issues

Lack of standardization across the studies and the stimulation methods are evident (Tables 5 and 6). Guidelines for sensory testing procedures have been presented [120–123], but standardized protocols, like the German Research Network on Neuropathic Pain (DFNS) [122–124] were not used in any of the studies. Aspects of data reproducibility and validity were only discussed in few studies [29,31,125].

## Conclusion

*ʻThe consistent failure to find an effect of naloxone on experimental pain in humans suggests that endorphin release did not occur during these procedures*ʼ [37].

This systematic review on endogenous opioid antagonism in physiological experimental pain models concludes that naloxone appears to have a demonstrable and relatively reliable effect in stress-induced analgesia (in all 7 studies) and repetitive transcranial magnetic stimulation (in all 3 studies). In all other pain models, both naloxone and naltrexone demonstrate a variable and unreliable effect.

## Supporting Information

S1 PRISMA Checklist (PDF)
